# Gecko Toe Pad-Inspired Robotic Gripper with Rapidly and Precisely Tunable Adhesion

**DOI:** 10.34133/research.0687

**Published:** 2025-04-23

**Authors:** Shuai Li, Hongmiao Tian, Xijing Zhu, Mingxin Liu, Xiangmeng Li, Jinyou Shao

**Affiliations:** ^1^ Shanxi Key Laboratory of Advanced Manufacturing Technology, School of Mechanical Engineering, North University of China, Taiyuan, Shanxi 030051, China.; ^2^ Micro- and Nano-technology Research Center, State Key Laboratory for Manufacturing Systems Engineering, Xi’an Jiaotong University, Xi’an, Shaanxi 710049, China.

## Abstract

Gecko-inspired dry adhesives have shown great potential in the field of robotics. However, there is still a large gap between current artificial adhesive-based grippers and natural geckos, especially in terms of precise and fast control of adhesion, which is an important capability for robotic gripper systems, since the targets to be gripped may vary in size and weight (including thin, fragile, soft, and deformable), and manipulation must be fast to meet high productivity requirements. Here, we propose a robotic gripper that is able to switch adhesion rapidly (in less than 0.5 s) to grasp and release objects of various sizes and weights (such as glass substrates, fragile silicon wafers, and deformable polyethylene terephthalate films) by mimicking the self-peeling behavior of gecko toe pads. The gripper retains the fast and stable manipulation of the conventional mechanical gripper, which is more reliable and has a higher load capacity than stimulus-responsive switchable adhesives. Systematic experimental and theoretical studies provide insights into the construction and analysis of the self-peeling model and mechanism to identify certain crucial parameters affecting the self-peeling behavior. Furthermore, a strategy for active adhesion control (i.e., precise adhesion modulation) is integrated by introducing a preset peeling angle *θ_B_*, providing the gripper with a quantitative criterion for adjusting the adhesion strength (0 to 82.77 kPa) according to the requirements of practical applications. The gripper has great potential to be an alternative end-operating gripper for robotic systems, opening an avenue for the development of robotic manipulation.

## Introduction

Natural evolution has produced many animal specialists with unique functions and survival skills that serve as inspiration for humans. In recent decades, numerous researchers have paid great attention to the adhesive pads of geckos because they have a remarkable climbing ability and can rapidly and precisely attach and detach from almost any surface by utilizing the van der Waals effects triggered by the micro/nanostructures on their toe surfaces [1–5]. Inspired by the bioadhesive properties of these biological structures, a variety of bioinspired adhesive materials with strong adhesion and good stability have been developed [6–14]. Such materials have shown promise in many application areas, including transfer printing [15–22], biomedical devices [23–25], climbing robots [26–28], and grippers [29–33]. Despite major advances in artificial adhesives, strategies to actively and rapidly control adhesion by mimicking the precise and switchable properties of gecko toe pads are still rare. Indeed, such strategies are of great importance for industrial systems, especially for robotic gripper systems in intelligent production lines, since the materials in the production lines may have different sizes and weights (even thin, fragile, soft, and deformable), and the manipulation must be fast to fulfill high productivity requirements.

The switchable adhesion of current bioinspired adhesives is mostly achieved by applying external stimuli such as heat, light, voltage, magnetic field, air pressure, and mechanical load to regulate the surface topography. However, these stimulus-response-based adhesives have some inherent limitations that restrict their applicability in high-speed robotic operation systems. For example, smart adhesives based on heat and light stimuli are generally slow to respond [34–37]. Although voltage/magnetic field/air pressure-activated smart adhesives have a relatively short response time, the complicated and costly additional devices such as high-voltage sources, magnetic control systems, and fluid channels need to be adapted to the robotic systems. More importantly, such methods achieve the release of objects to a certain extent through the combined effects of surface topography regulation and object gravity, which means that it is difficult or time-consuming for them to handle thin and soft objects [30,31,38–42]. It is noteworthy that the 2 magnetically actuated smart adhesives proposed by Wang et al. [43] and Linghu et al. [44] are able to handle thin, fragile, or heavy objects such as wafers, spherical lampshades, and aluminum plates within 0.5 s due to their special bionic structure designs, which have the potential for application in deterministic assembly and industrial or robotic manipulation. An approach based on mechanical load, such as elastic buckling-based release, has the advantages of manipulating rigid objects, e.g., thick glass substrates and cell phones, but may be problematic for thin and fragile objects due to the high preload [45–47]. Existing stimulus-response-based adhesives are limited in terms of response time and precise control, and there is a wide gap between them and geckos.

In fact, the rapid and precise attachment/detachment of gecko toe pads benefits from their self-peeling and back-scrolling mechanism [48,49], but it has not been fully exploited and reproduced, which has been practically used in only a few applications, such as the detachment phase of the bioinspired robot (Stickybot) developed by Kim et al. [27] and the released state of the 3-legged clamps proposed by Zhou et al. [50] and Tian et al. [51]. In addition, some researchers have also developed a limited number of stimulus-responsive grippers with potential applications by mimicking the self-peeling of gecko toe pads [52–54]. For example, Shahsavan et al. [52] developed a multilegged gecko gripper with a thermally induced self-peeling capability by integrating gecko-inspired adhesives into hybrid nematic side-chain liquid crystal polymer cantilevers. The gripper is capable of handling (picking and placing) a thin 4-inch silicon wafer with a mass of ≈9 g, but it is time-consuming and the load-bearing capacity is weak due to the flexibility of the liquid crystal polymer cantilevers. Zhang et al. developed a multilayer, self-peeling, switchable dry/wet adhesive by combining thermally responsive hydrogel layers, gecko foot-inspired mushroom structures, and mussel-inspired copolymer adhesive coatings. The adhesive is able to grip/release objects through a thermally responsive curving behavior, but it is also time-consuming and has a low load-bearing capacity [54]. Therefore, the development of a reliable robotic gripper with rapidly and precisely tunable adhesion based on the self-peeling mechanism of gecko toe pads is still a scientific and technical challenge.

It is known that mechanical grippers are generally faster, more stable, and more accurate than stimulus-responsive grippers for object manipulation in intelligent production lines. However, mechanical grippers are limited to the traditional multifinger designs that are unable to handle thin and fragile objects [33,55,56]. The aim of this work is to develop a new bioinspired robotic gripper that retains the fast and stable actuation and outstanding load-bearing capacity of the traditional mechanical gripper while replacing the multifinger configuration with an actively self-peeling design that mimics the gecko toe pad. Such a gecko toe pad-inspired robotic gripper is capable of handling materials of various sizes and weights quickly and accurately (in less than 0.5 s), even thin, fragile, soft, and deformable objects. Systematic experimental and theoretical studies provide insights into the construction and analysis of the self-peeling model and mechanism to determine some crucial parameters that influence the self-peeling behavior. In addition, a precise adhesion modulation of the gripper is proposed and demonstrated, which presents a quantitative criterion for the gripper to adjust the adhesion strength according to the requirements of practical applications and has never been reported in other publications. It is envisioned that such a bioinspired robotic gripper could have promising applications and has great potential to become a new end-operating gripper for robotic systems.

## Results

### Design of the robotic gripper with precisely tunable adhesion

**Fig. 1.**
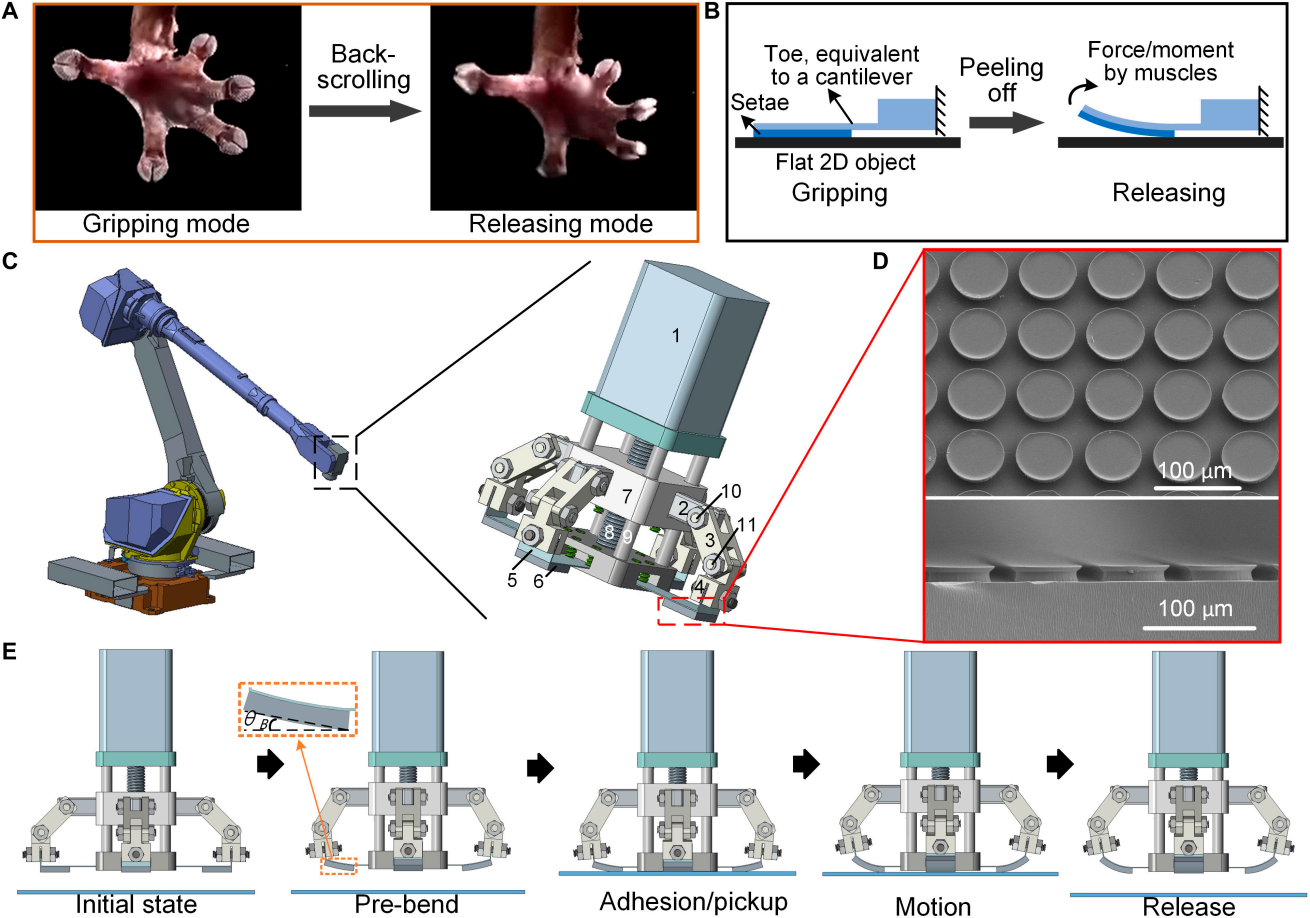
Schematics of the bionic background, structure and operating mechanism of the robotic gripper with precisely tunable adhesion. (A) Optical images of gecko toes fully extended in gripping and back-scrolled in releasing modes. (B) Peeling-off model extracted from the back-scrolling behavior of gecko toes. (C) A 3-dimensional (3D) assembly of the proposed gripper, which has the potential to become a new end-operating gripper of the robot. 1, motor; 2, 3, and 4, connecting rods; 5, stainless steel sheet; 6, adhesive film; 7, slider; 8, screw; 9, guide shaft; 10 and 11, hinges. (D) Scanning electron micrographs of the bioinspired adhesives. Each microadhesive structure measures 32.5 μm in radius. (E) Operating mechanism of the gripper. 2D, 2-dimensional.

Particular configurations of gecko toes fulfill essential functions in regulating adhesion during their locomotion. As shown in Fig. 1A, in the gripping mode, the seta arrays on the gecko toes are pushed and dragged against the mating surfaces by the gecko muscles to establish intimate contact [57–59]. In the releasing mode, with the heel of the palm pad as the fulcrum, the toes curl and scroll upward and away from the surface, driven by the muscles, to rapidly reduce the high adhesion/friction, for perpendicular peeling off of the seta arrays from the substrates [4]. The back-scrolling motion of the toes at any instant during detachment concentrates the detachment force on only a small portion of all attached setae [1].

A peeling-off model is extracted from the back-scrolling behavior of the toe pads, as shown in Fig. 1B. The palm and the toe of the gecko correspond to the fulcrum and the cantilever, respectively. After the seta arrays make full contact with the mating surface, e.g., a flat 2-dimensional object, the force or moment of the gecko muscles actuates the toe (cantilever) to bend upward to facilitate release. Motivated by the peeling-off model, a gecko toe pad-inspired robotic gripper with precisely tunable adhesion was developed, which has great potential for a new end-operating gripper of the robot (Fig. 1C). The gripper with an overall size of about 65 mm × 70 mm consists of 3 parts: the drive module (1, motor; 7, slider; 8, screw; 9, guide shaft), the link module (2, 3, and 4, connecting rods; 10 and 11, hinges), and the adhesion module (5, stainless steel sheet; 6, adhesive film), in which the adhesive film is fabricated by the technology of double exposure, material filling, and curing shrinkage described in our previous research (Fig. 1D) [60]. It should be noted that if adopting scaled-up/down assembly parts, the overall size of the gripper is also scaled up/down, which can be applied to different manipulation conditions.

The operating mechanism of the gripper is shown in Fig. 1E. First, the gripper is set to the initial state. Then, a preset peeling angle *θ_B_* (≥0°) is introduced by pre-bending the adhesion module pulled by the motion of the motor (the rotary motion of the motor is converted into a linear motion of the slider by the screw) to precisely modulate the contact area and further adhesion performance between the adhesion module and the substrate. After the pre-bent gripper approaches and contacts the substrate, adhesion occurs, and the gripper can pick up or transfer the target object. Notably, as the current adhesion module is constrained by the link module, the gripper is able to demonstrate a high load capacity. When the object reaches its destination and has to be released, the adhesion module bends upward due to the motion of the motor. The adhesion force decreases during the motion until the target object achieves release. At the moment, the adhesion module is in the phase of elastic deformation and not plastic deformation due to the high yield stress of the adhesion module. Therefore, when the reset signal is transmitted to the motor, both the gripper and the adhesion module are quickly reset to prepare for the next manipulation. From the operating mechanism, it is plausible that this mechanical gripper has superior abilities to reliably and rapidly grip and release objects compared to other bioinspired grippers that rely on external stimuli such as heat and light.

### Critical design parameters of the gripper

**Fig. 2.**
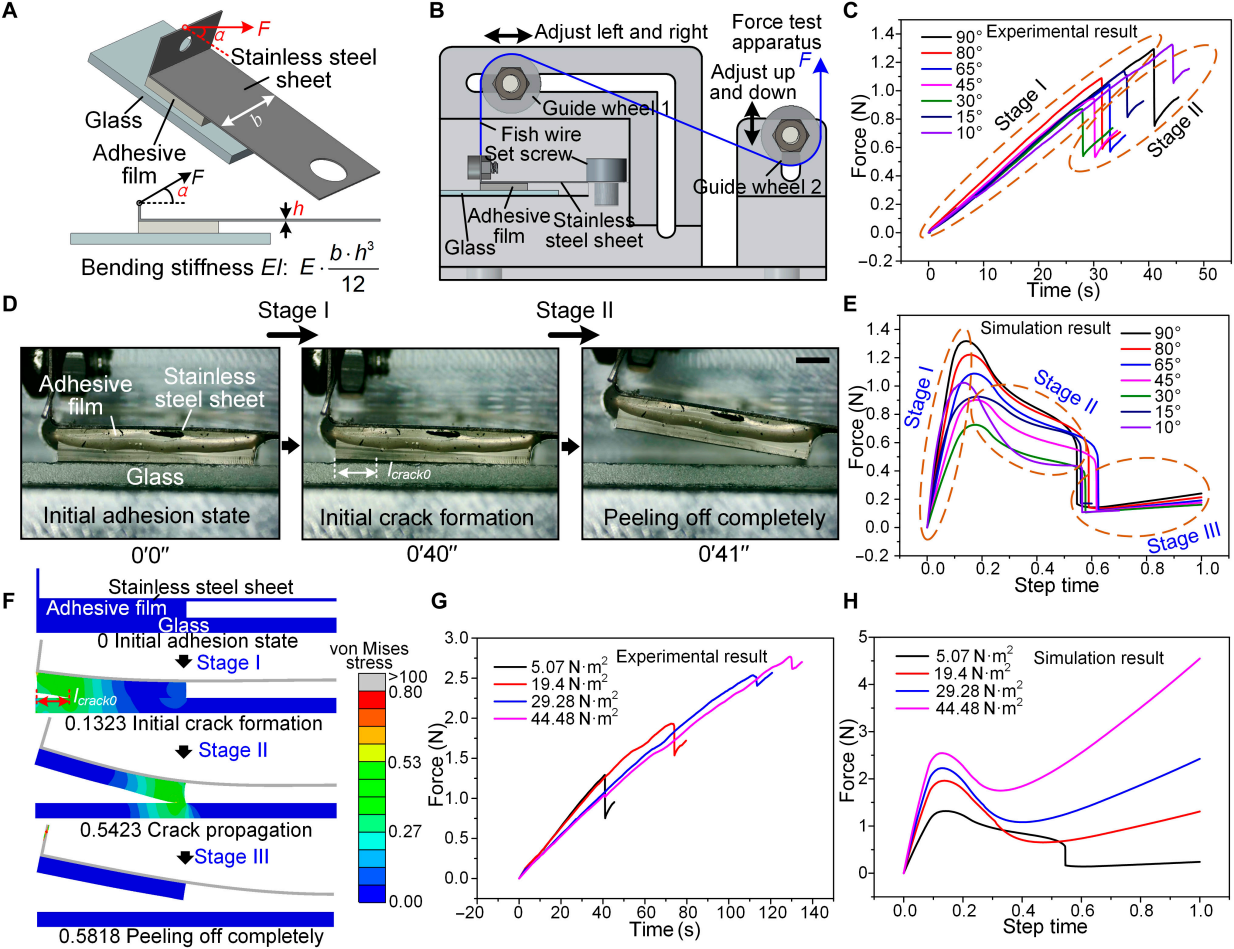
Analysis of the critical design parameters of the gripper. (A) Two main parameters (the initial pulling angle *α* of the adhesion module and the bending stiffness *EI* of the stainless steel sheet) that affect *F_max_*. (B) Schematic diagram of the homemade test device. (C) Experimental curves of the force as a function of time at different *α* values. (D) Peeling process of the adhesive module recorded with a charged-coupled device (CCD) camera. The scale bar corresponds to 1 mm. (E) Variation curves of the peeling force with the step time for different *α* values during the peeling simulation. (F) Peeling process recorded with the finite element analysis (FEA) simulation, with the cloud atlas representing the von Mises stress distribution. (G) Force–time test curves at different *EI* values. (H) Variation curves of peeling force versus step time at different *EI* values during the peeling simulation.

There are some basic criteria that should be met to enable gripping and releasing. For gripping, the adhesion strength of the adhesive patches need to be high enough to withstand the force of gravity acting on the lifted object. For releasing, the peeling force of the adhesion module triggered by the motion of the motor must be higher than the adhesion of the patches but as low as possible to minimize the motor loads (Fig. S1). Since the average adhesion strength of the adhesive patches is high enough (up to 205 kPa, based on our previous research), the maximum peeling force *F_max_* for achieving release is actually the only design factor of interest in this work. As can be seen in Fig. 2A, the peeling force is mainly determined by the initial pulling angle *α* and the bending stiffness *EI* of the adhesion module. In addition, because the elastic modulus of the adhesive film (~2.5 MPa) is much lower than that of the stainless steel sheet (190 GPa), the *EI* of the adhesive film has only a minor influence on the peeling force. Therefore, the *α* of the adhesion module and the *EI* of the stainless steel sheet are the 2 most important parameters influencing *F_max_*. To investigate the influence of these 2 parameters on *F_max_*, a homemade test device was built as shown in the schematic diagram in Fig. 2B. The beginning of the stainless steel sheet is fixed with a grub screw, and the end is connected to the fish wire with good resilience. The end of the fish wire is connected to the load cell of the force test apparatus via guide wheels 1 and 2, which can be adjusted to the left/right or up/down to vary *α*. After the adhesive film is in full contact with the flat glass by applying a sufficient preload, the stainless steel sheet is pulled upward at a certain speed of 5 mm/min until the adhesive film is completely separated from the glass. In the meantime, the varying force is recorded by the load cell through the transmission of the fish wire.

Figure 2C shows the experimental curves of force versus time at different *α* values, where the sizes of the stainless steel sheet and adhesive film are 18 mm × 8 mm × 0.2 mm and 10 mm × 8 mm × 1.1 mm, respectively. For all *α*, the force initially increases with increasing time until *F_max_* is reached; this phase is referred to as stage I. Then, the force decreases sharply until it increases again (Fig. S2); this phase is referred to as stage II. The specific explanations for stages I and II can be exploited by the peeling-off process of the adhesion module recorded by a charged-coupled device (CCD) camera, as shown in Fig. 2D and Movie S1. The initial adhesion state, i.e., when the adhesive film is in full contact with the flat glass, is denoted as 0 s. As the applied force increases, an initial crack *l_crack0_* is formed at 40 s due to the equilibrium between the applied energy and the work of separation, and at the same time *F_max_* is reached, which corresponds to stage I in Fig. 2C. Subsequently, the energy balance is broken, peeling occurs rapidly, and the peeling force drops sharply until the adhesive film is completely peeled from the glass. At this point (41 s), when the adhesion module is pulled further upward, the stainless steel sheet deforms further and the peeling force increases again, which corresponds to stage II in Fig. 2C.

To better understand the separation processes for different *α* values, the corresponding finite element analysis (FEA) peeling model was built and simulated based on the theory of the interfacial cohesion zone (Fig. S3). The details are provided in Note S1. The variation curves of peeling force with step time for different *α* values during the peeling simulation are shown in Fig. 2E. Similar to the experimental results, the peeling force for all *α* initially increases with the step time until the *F_max_* value is reached, and this stage is noted as stage I. Since the fracture energy is set at 0.2 mJ, the crack propagation in Fig. 2E takes a certain step time until the adhesive film is completely peeled off the glass, which is noted as stage II. The peeling force then drops sharply until it increases again due to the continued deformation of the stainless steel sheet, which is recorded as stage III. Figure 2F, similarly to Fig. 2D, shows the entire peeling evolution process to explain the 3 stages more intuitively (Movie S2), with the cloud atlas representing the von Mises stress distribution. The initial adhesion state is recorded as step time 0. When the analysis step time reaches 0.1323 (stage I), the initial crack *l_crack0_* is generated and *F_max_* is reached, which is demonstrated by the variation curve of the scalar stiffness degradation for cohesive surfaces (CSDMG) of the first damage node of the adhesive film as a function of the step time (Fig. S5). Subsequently (stages II and III), the crack continues to propagate until the adhesive film is completely detached from the glass (0.5818). The evolution of the maximum normal stress of the adhesive film and the energy of each component (external work, strain energy, and damage dissipation energy) during the peeling process can also be an intuitive explanation for the initiation and propagation of interfacial cracks, as illustrated in Figs. S6 and S7.

The other parameter, the bending stiffness *EI* of the stainless steel sheet, affects *F_max_* by influencing the deformability of the adhesion module. The expression for *EI* is as follows:

$$\mathrm{EI}=E\cdot \frac{b\cdot h^{3}}{12}$$ (1)

where *E* and *I* represent the elastic modulus and the area moment of inertia of the stainless steel sheet, respectively. The parameters *b* and *h* are the width and thickness of the stainless steel sheet, respectively, and *b* = 8 mm and *h* is set to 0.2, 0.35, 0.43, and 0.53 mm (Fig. S8) to obtain different *EI* values of 5.07, 19.4, 29.28, and 44.48 N·m^2^, respectively. Figure 2G shows the force–time test curves at different *EI*. Similar to the results in Fig. 2C, for all *EI*, the peeling force gradually increases as the load is applied until it reaches *F_max_*. After that, the peeling force decreases sharply (Fig. S9) and the adhesive film is completely separated from the glass substrate. However, due to the continued deformation of the stainless steel sheet caused by the sustained upward pull, the peeling force increases again. In addition, the peeling model based on the interfacial cohesive zone theory was also built and simulated to investigate the separation processes for different *EI* values (Fig. S10). Figure 2H shows the variation curves of peeling force versus step time at different *EI* values during the peeling simulation. Similar to the experimental results, the peeling force for all *EI* initially increases with the step time until *F*_max_ is reached and the initial crack *l_crack0_* is generated. Subsequently, the crack propagates and the peeling force decreases. The crack propagation takes a certain step time to completely detach the adhesive film from the glass, as the fracture energy is set at 0.2 mJ. Finally, as the stainless steel sheet deforms further, the peeling force increases again. In addition, thinner stainless steel sheets (e.g., 0.15 mm) were also used in peeling tests. However, they are subject to considerable plastic deformation or are very susceptible to bending damage.

### Peeling mechanism

The object of the general theoretical peeling model is a single adhesive film [4,52,61,62]. However, the adhesion module we propose consists of 2 parts: adhesive film and a stainless steel sheet. The introduction of the stainless steel sheet increases the complexity of the theoretical model, so that the general theoretical peeling model is no longer applicable.

**Fig. 3.**
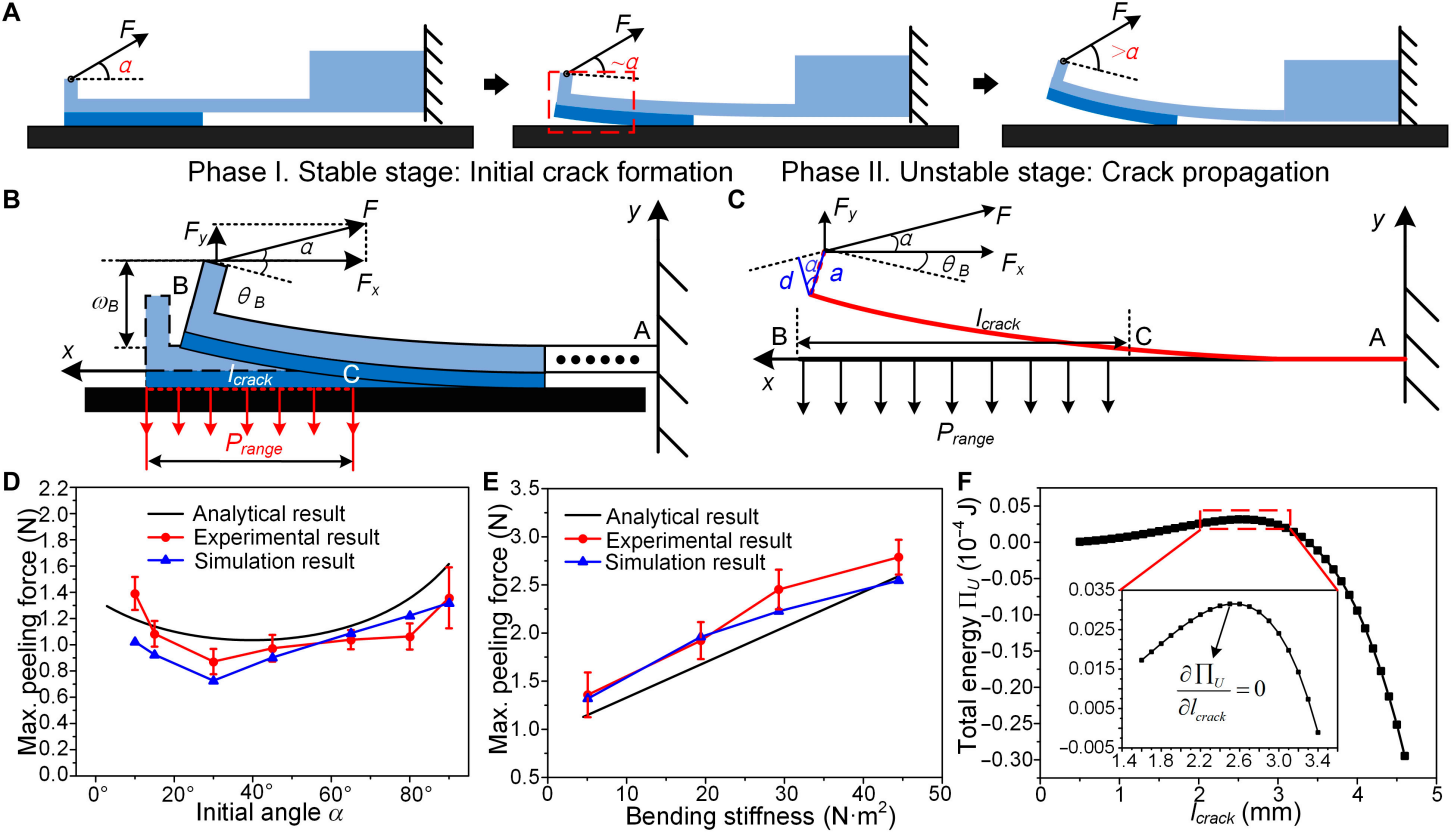
Analysis of the peeling mechanism. (A) Schematic diagram of the whole peeling process, consisting of phase I, the stable stage from initial adhesion to initial crack formation, and phase II, the unstable stage with rapid crack propagation. (B) Mechanical model of the stable stage. (C) Simplified free-body diagram of the model. (D) Comparison between numerical analysis and experimental and simulation results of *F_max_* vs. *α*. (E) Comparison between numerical analysis and experimental and simulation results of *F_max_* vs. *EI*. (F) Evolution of the total energy П*_U_* in the crack region (0.5 to 4.5 mm) near the initial crack *l_crack0_*. Error bars represent the SD for *n* = 3.

From the above experiments and simulations, it can be seen that the whole peeling process can be divided into 2 phases, namely, phase I, the stable stage from the initial adhesion state to the initial crack formation, and phase II, the unstable stage with rapid crack propagation, as shown in Fig. 3A. Among them, the deformation of the stainless steel sheet during the stable stage is small, that is, the small deflection deformation of the cantilever beam under the action of the peeling force *F* and the adhesion force *P_range_*, so the initial pulling angle *α* changes very little and can be regarded as constant, so the mechanism can be analyzed by using the small deformation theory of the cantilever beam based on the Euler–Bernoulli beam theory [63]. In addition, the stainless steel sheet in the unstable stage is subjected to large deformation by the peeling force *F* and the adhesion force *P_range_*, and *α* varies greatly, which is a large deflection deformation model. However, this stage has already passed the point at which *F_max_* is reached and is no longer the focus of our attention, so it is not analyzed in this work.

The mechanical model of the stable stage taken from Fig. 3A is shown in Fig. 3B, where the cantilever support point is noted as point A, the end of the adhesion module as point B, and the initial crack as point C. The adhesion module is subjected to 2 loads, the peeling force *F* and the adhesion force *P_range_*. At the moment of formation of the initial crack *l_crack0_*, point B generates a deflection and an angle of rotation of *ω_B_* and *θ_B_*, respectively. *α* is considered constant. Figure 3C shows the simplified free-body diagram of the model. Decomposing the peeling force *F* into its *x* and *y* components yields

$$\begin{array}{l} F_{x}=F\cdot \text{cos}\left(\alpha \right)\\ F_{y}=F\cdot \text{sin}\left(\alpha \right) \end{array}$$ (2)

The length of the end of the stainless steel sheet is denoted by *a*, and its component in the direction perpendicular to *F* is denoted by *d*, and *d* = *a*·cos(*α*).

According to the mechanics of materials [64], the second-order differential equation for the deflection curve of the adhesion module is as follows:

$$\frac{d^{2}\omega }{{\mathrm{dx}}^{2}}=\frac{M\left(x\right)}{\mathrm{EI}}$$ (3)

where *EI* approximates the bending stiffness of the stainless steel sheet, since the bending stiffness of the stainless steel sheet is much greater than the bending stiffness of the adhesive film. *M*(*x*) is the bending moment acting on the adhesion module, which consists of 2 components:

$$M\left(x\right)=M\left(F\right)-M\left(P_{\text{range}}\right)$$ (4)

where *M*(*F*) and *M*(*P_range_*) are the bending moments generated by *F* and *P_range_*, respectively.

The peeling force *F* and the adhesion force *P_range_* are then decoupled; i.e., the superposition method in mechanics of materials is used to investigate the action of both on the adhesion module separately (Fig. S11). The detailed calculation process is described in Note S2. Subsequently, by superimposing the angle of rotation and the deflection at point B under the action of *F* and *P_range_*, respectively, the total angle of rotation and the total deflection at point B can be determined as follows:

$$\theta_{B}=\frac{F\cdot l_{\text{crack}}}{\mathrm{EI}}\left(a\text{cos}\alpha +\frac{l_{\text{crack}}\cdot \text{sin}\alpha }{2}\right)-\frac{P_{\text{range}}\cdot b\cdot l_{\text{crack}}^{3}}{6\mathrm{EI}}$$ (5)

$$\omega_{B}=\frac{F\cdot l_{\text{crack}}^{2}}{2\mathrm{EI}}\left(a\text{cos}\alpha +\frac{2l_{\text{crack}}\cdot \text{sin}\alpha }{3}\right)-\frac{P_{\text{range}}\cdot b\cdot l_{\text{crack}}^{4}}{8\mathrm{EI}}$$ (6)

When the initial crack *l_crack0_* is generated and the peeling force reaches its maximum, the system is in an unstable equilibrium; i.e., the stable stage passes into the unstable stage, so that the crack propagation during the transition from the stable to the unstable stage obeys Griffith’s energy criterion for fracture [65]; the energy method can be used to solve for the maximum peel force *F_max_*. The total energy of the system П*_U_* includes the work *W_F_* performed by the peeling force *F*, the elastic strain energy *U_ε_* stored in the adhesion module during the stable stage, and the free surface energy *U_Г_* required to create a new surface at the adhesion interface [6,66], i.e.,

$$\Pi_{U}=-W_{F}+U_{\varepsilon }+U_{\Gamma }$$ (7)

where $U_{\varepsilon }=U_{b,g}+U_{s,t}$, and *U*_*b*,*g*_ and *U*_*s*,*t*_ represent the bending strain energy stored in the stainless steel sheet and the tensile strain energy stored in the adhesive film, respectively. Detailed calculation processes for *W_F_*, *U*_*b*,*g*_, *U*_*s*,*t*_, and *U_Г_* are provided in Note S2.

According to Griffith’s energy criterion for fracture [65], the total energy of the system and the crack length *l_crack_* should satisfy an energy release rate of 0 [67–69], i.e.,

$$\frac{\partial \Pi_{U}}{\partial l_{\text{crack}}}=\frac{\partial }{\partial l_{\text{crack}}}\left(-W_{F}+U_{\varepsilon }+U_{\Gamma }\right)=0$$ (8)

The expression for *F_max_* can be obtained by solving Eq. 8 as follows:

$$F_{\max}=\frac{b\cdot P_{\text{range}}^{2}\cdot l_{\text{crack}}^{4}+12\mathrm{EI}\cdot W_{\mathrm{ad}}}{2P_{\text{range}}\cdot \text{sin}\alpha \cdot l_{\text{crack}}^{3}+2P_{\text{range}}\cdot a\cdot \text{cos}\alpha \cdot l_{\text{crack}}^{2}}$$ (9)

By substituting the parameter values from Table S2 into Eq. 9, the results of the numerical analysis of the variation of *F_max_* with *α* can be obtained and compared with the experimental and simulated *F_max_* from Fig. 2C and E, as shown in Fig. 3D. It is obvious that the 3 results are in good agreement with each other. However, the minimum value of *F_max_* in the numerical analysis is obtained at *α* = 39.53°, which is slightly different from the minimum value obtained at *α* = 30° in the experiment and simulation. This difference can be explained by the fact that the experimental and simulated results do not have dense data points. In addition, by setting the initial pulling angle *α* to 39.53° and taking the bending stiffness *EI* as the independent variable, the results of numerical analysis of *F_max_* with *EI* can be obtained and compared with the experimental and simulated *F_max_* from Fig. 2G and H, as shown in Fig. 3E. It can be seen that the 3 results agree with each other and are all substantially linearly correlated with *EI*, with almost identical slopes. In addition, since a larger maximum peeling force increases the load on the motor during the peeling process according to Fig. S1, which has a detrimental effect on the service life of the motor, the thickness *h* of the stainless steel sheet and the initial pulling angle *α* of the gripper are set to 0.2 mm and 39.53°, respectively, to minimize the load on the motor.

In addition to the *F_max_* required for detachment, the evolution of the energy of the components and the total energy of the system is also important to fully characterize the peeling process. The values for *F_max_* and the initial pulling angle *α* are set to 1.035 N and 39.53°, respectively (from the results of the numerical analysis), and the evolution of the energy of each component with *l_crack_* during the stable stage is plotted, as shown in Fig. S12. With increasing *l_crack_*, *W_F_* gradually increases, and *U*_*b*,*g*_, *U*_*s*,*t*_, and *U_Г_* also gradually increase and have signs opposite to that of *W_F_*, indicating that *W_F_* is converted into the latter 3 components and *U*_*b*,*g*_ accounts for most of the energy conversion. Figure 3F demonstrates the evolution of the total energy П*_U_* in the crack region (0.5 to 4.5 mm) near the initial crack *l_crack0_*, and the inset shows a local enlargement of the curve in the red dashed box. It can be seen that when *l_crack_* < *l_crack0_*, then П*_U_* > 0, which means that *W_F_* < *U_ε_* + *U_Г_*, and the energy release rate $\partial \Pi_{U}\;/\;\partial l_{\text{crack}}$ is positive at this time (Fig. S13). According to the principle of minimum energy [70], it is difficult to propagate the crack at this time to ensure that the total energy is minimized. If *l_crack_* > *l_crack0_*, the total energy of the system, П*_U_*, decreases rapidly and eventually becomes less than 0, i.e., *W_F_* > *U_ε_* + *U_Г_*, and the energy release rate at this point is negative. The crack must propagate rapidly to reduce the total energy. In other words, the peeling process enters an unstable stage.

### Precise adhesion modulation of the gripper

**Fig. 4.**
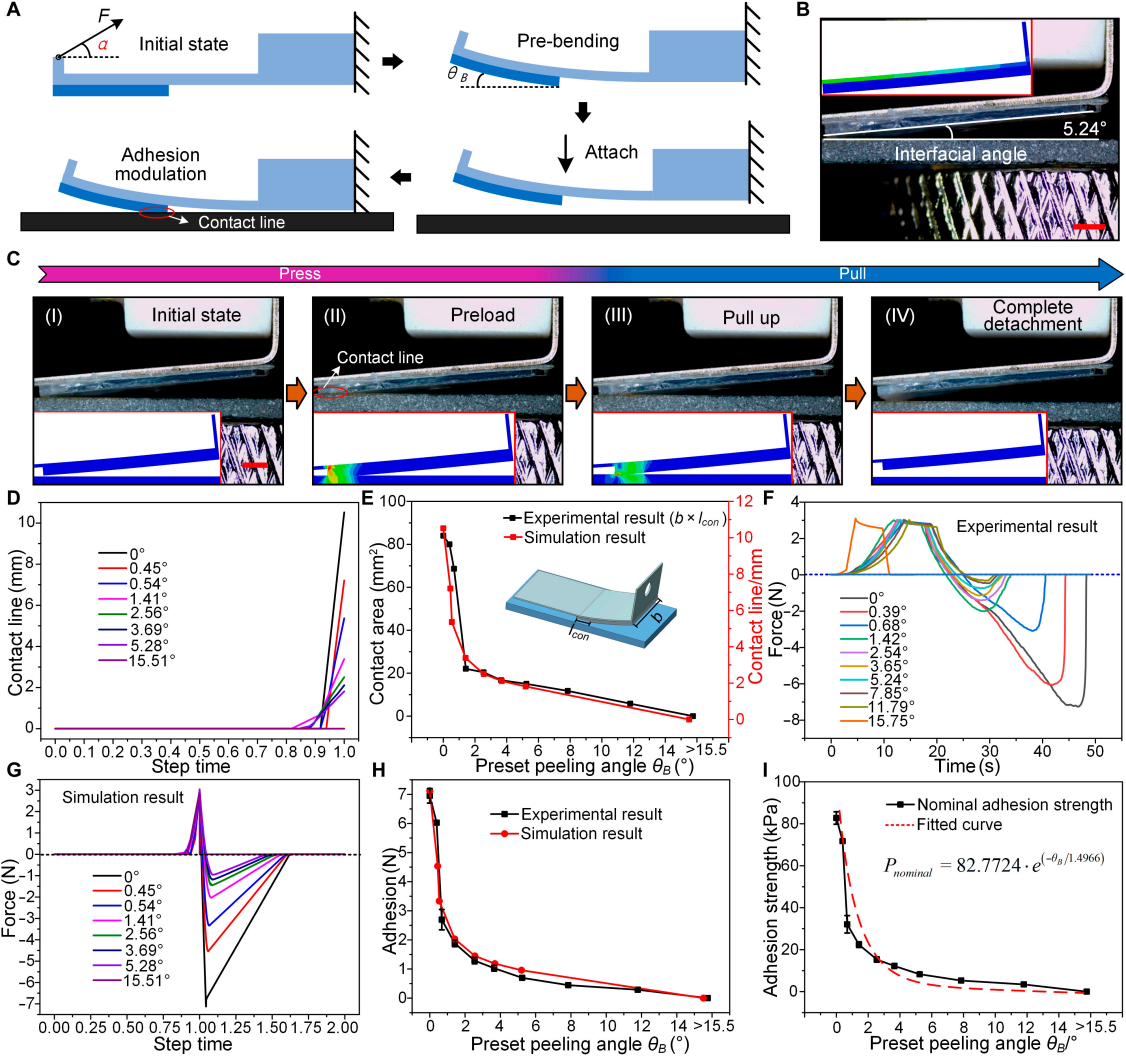
Precise adhesion modulation of the gripper. (A) Schematic diagram of the precise adhesion modulation of the gripper, consisting of the processes of pre-bending and precise adhesion modulation. (B) Preset peeling angle *θ_B_* (5.24°) introduced by the motion of the motor. The numerical result is consistent with that of the experimental preset peeling angle. The scale bar corresponds to 1 mm. (C) Dynamic behavior of the adhesion module with a *θ_B_* of 5.24° upon contact and detachment from a glass surface. The numerical result is consistent with that of the experimental dynamic behavior. The scale bar corresponds to 1 mm. (D) Evolution of the contact line for different *θ_B_* values at an identical preload (3 N). (E) Comparison between the contact line in the simulation and the actual contact area. (F and G) Experimental and simulated curves of the force as a function of time/step time at different *θ_B_* values. (H) Comparison between experimental and simulation results of the maximum adhesive force as a function of *θ_B_*. (I) Exponential regression of the nominal adhesion strength *P_nominal_* against *θ_B_*. Error bars represent the SD for *n* = 3.

Based on the peeling mechanism, crack propagation (i.e., the gradual increase in the peeling angle *θ_B_*) can lead to a decrease in contact area and adhesion. This phenomenon indicates that the gripper is capable of precisely modulating adhesion when a preset *θ_B_* is introduced into the adhesion module. As illustrated in Fig. 4A, the initial adhesion module with an *α* of 39.53° and an *h* of 0.2 mm (from the results of numerical analysis) is pre-bent by the motion of the motor to introduce a preset peeling angle *θ_B_* (Fig. 4B and Figs. S14 and S15). Then, the pre-bent gripper approaches and contacts the substrate. It should be noted that the contact area between the adhesion film and the substrate at this point is not complete due to the existence of *θ_B_* and can be precisely modulated by adjusting *θ_B_* to achieve further modulation of adhesion during pulling-up. Figure 4C shows the dynamic behavior of the adhesion module with a *θ_B_* of 5.24° contacting and detaching from a glass surface. The corresponding dynamic motions recorded by the CCD camera and FEA, which are in good agreement, can be seen in Movies S3 and S4. The details of the simulation model are provided in Note S3.

Figure 4D displays the evolution of the contact line for different *θ_B_* values with an identical preload (3 N) in the simulation, where the contact line at 0 to 0.88 s remains at 0 since there is no contact between the adhesion module and the substrate. From 0.88 s, the contact line becomes increasingly larger with increasing time, up to 1 s. At this point, the adhesive module begins to detach from the substrate, which corresponds to the snapshot with the maximum contact area. For further investigation, the maximum contact line at different *θ_B_* values is extracted from Fig. 4D and plotted in Fig. 4E (red line) in comparison to the actual contact area (i.e., actual contact line *l_con_* × width of adhesion module *b*, black line). It can be seen that the experimental and simulation results are in good agreement; i.e., the contact line/area first decreases rapidly in the *θ_B_* range of 0° to 1.42°, then decreases slowly in the *θ_B_* range of 1.42° to 15.5°, and finally decreases to zero when *θ_B_* is larger than about 15.5° because there is no more contact between the adhesion film and the substrate. The corresponding experimental and simulated curves of force as a function of time/step time at different *θ_B_* values are shown in Fig. 4F and G, respectively. Similar to the variations of the contact line/area, the adhesive force in both the experiments and the simulation initially decreases with increasing *θ_B_* and then decreases to zero when *θ_B_* is larger than about 15.5°, as there is no longer any contact between the adhesion film and the substrate. Figure 4H summarizes and plots the curves of the maximum adhesive force at different *θ_B_* values from Fig. 4F and G. It can be seen that the experimental and simulated results agree well and the valid modulation range of *θ_B_* on the adhesive force is 0° to 15.5°.

In addition, an intuitive illustration of the nominal adhesion strength *P_nominal_* as a function of *θ_B_*, where *P_nominal_* is defined as $F_{\text{adhesion}}\;/\;A_{\theta_{B}=0{}^{\circ}}$ (where $F_{\text{adhesion}}$ is the maximum adhesive force in Fig. 4F and $A_{\theta_{B}=0{}^{\circ}}$ is the contact area at *θ_B_* = 0°, which is equal to 84 mm^2^), is shown in Fig. 4I. It can be seen that *P_nominal_* exhibits the same evolution trend as the adhesive force in Fig. 4H; i.e., *P_nominal_* decreases from 82.77 kPa at 0° to 0 kPa at 15.5° and is maintained at 0 kPa when *θ_B_* is greater than about 15.5°. To evaluate the precise adhesion modulation of the gripper, an exponential regression function $P_{\text{nominal}}=82.7724\cdot e^{\left(-\theta_{B}/1.4966\right)}$ is used to fit the curve (red dashed line), which presents a quantitative criterion for the gripper to adjust the adhesion strength by regulating *θ_B_* according to the requirements of practical applications.

### Applications of the intelligent robotic system

**Fig. 5.**
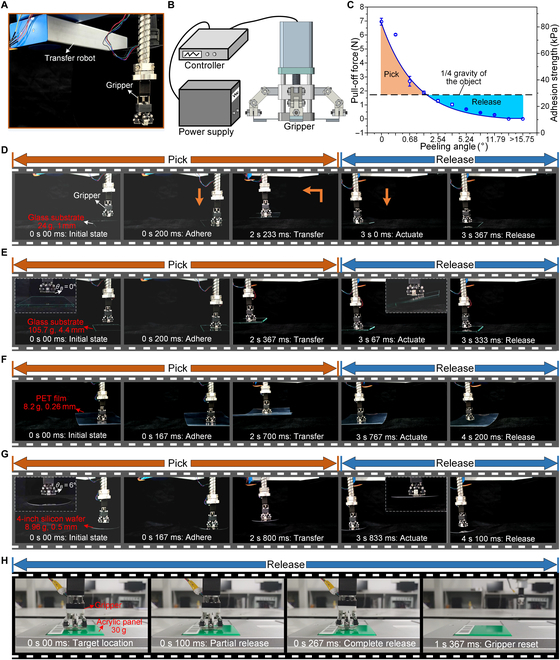
Demonstration of the robotic system in the fast and precise manipulation of materials of various sizes and weights. (A) Robotic system consisting of a transfer robot and a gripper (with an *α* of 39.53° and an *h* of 0.2 mm). (B) Electrical diagram of the gripper. (C) Reliable gripping and releasing regions of the gripper. (D to G) Applications of the robotic system in the automatic manipulation of thin, fragile, soft, and deformable objects in advanced laboratories. (H) Application of the robotic system in the manipulation of a commercial acrylic panel in an intelligent production line. Error bars represent the SD for *n* = 3. PET, polyethylene terephthalate.

The above discussions reveal the critical design parameters, the peeling mechanism, and the advantages of precisely tunable adhesion of the gripper. Figure 5 illustrates the promising applications of the gripper in the fast and precise manipulation of materials of various sizes and weights, such as thin, fragile, soft, and deformable objects. For this purpose, a robotic system consisting of a transfer robot and a gripper (with an *α* of 39.53° and an *h* of 0.2 mm) was constructed, as shown in Fig. 5A. The corresponding electrical diagram of the gripper is shown in Fig. 5B. Specifically, the voltage supplied by the power supply is conducted to the controller, which can adjust the speed and distance of the motor. The gripper would respond quickly to the signal triggered by the controller to perform the manipulation (pre-bend, release or reset). In the gripping mode, the adhesive force of the gripper must be greater than one-fourth of the object’s gravity to ensure a reliable grip, which corresponds to the orange area (i.e., the reliable gripping area) as shown in Fig. 5C. It should be noted that in this reliable gripping area, the gripper is able to adjust the adhesion force by regulating *θ_B_* according to the weight of the target object to shorten the subsequent release reaction time as much as possible. In the releasing mode, the object can be reliably released once the adhesion force of the gripper can no longer overcome one-fourth of the gravity of the object with increasing *θ_B_*, which corresponds to the blue area (i.e., the reliable releasing area) as shown in Fig. 5C.

Figure 5D to G demonstrate the applications of the robotic system in the automatic manipulation (i.e., gripping, transferring, and releasing) of thin, fragile, soft, and deformable objects (such as glass substrates, silicon wafers, and polyethylene terephthalate [PET] films) in advanced laboratories (Movies S5 to S8). The manipulation process is the same as in Fig. 1E, in which *θ_B_* in the manipulation of glass substrates (with weights of 24 and 105.7 g and thicknesses of 1 and 4.4 mm) and PET film (with a weight of 8.2 g and a thickness of 0.26 mm) is set to 0° due to the relatively high weight of the glass substrates and the deformability of the PET film, and *θ_B_* is set to 6° when manipulating a 4-inch silicon wafer (with a weight of 8.96 g and a thickness of 0.5 mm) due to the relatively low weight. It can be seen that for all objects, the manipulation cycle time is less than 4.1 s and can be further reduced by increasing the robot’s operating speed, which is sufficient for robotic manipulation. In addition, the release time of the target object during the entire cycle time is the most critical parameter and deserves to be emphasized. Because of the fast response to the motion of the motor, the releasing times for thin and thick glass substrates, PET film, and silicon wafer are 367, 267, 433, and 267 ms, respectively, which has evident advantages compared to the approaches of smart materials triggered by external stimuli, such as thermal and light-based systems [29,34,35,37,71]. It should be noted that the relatively short releasing time for silicon wafer demonstrates the discussion in Fig. 5C; i.e., the gripper can adjust the adhesion force by regulating *θ_B_* according to the weight of the target object to shorten the releasing response time. Furthermore, due to its high load capacity, the proposed gripper can also handle some relatively rigid and large objects, such as a ceramic dish (490 g), an iPad (460 g), a glass substrate (433 g), and a cell phone (245 g), as shown in Fig. S17.

In addition to applications for manipulating objects in advanced laboratories, the proposed gripper also demonstrates its application in an intelligent production line. Specifically, an industrial robot with a gripper integrated at the end was used to repeatedly manipulate commercial acrylic panels (weighing 30 g) in an intelligent production line (Movie S9). Figure 5H shows the rapid release process of the acrylic panel. When the acrylic panel reaches the target position (marked as 0 s), the motor starts to move, triggering the releasing behavior of the adhesion module. As the motor continues to move, the acrylic panel is partially released at a time of 100 ms and completely released at a time of 267 ms. Finally, the gripper is reset at a time of 1 s 367 ms. It can be seen that the releasing time for commercial acrylic panel is only 267 ms, which is comparable to the response time of mammalian skeletal muscles. More importantly, the gripper still exhibits a high adhesion after 10,000 cycles of picking and placing, which proves its robustness, as shown in Fig. S18.

These specific examples clearly demonstrate the rapidly and precisely tunable adhesion of the proposed gripper, which is beneficial for robotic manipulation with high tempo.

## Discussion

In summary, we propose a novel gecko toe pad-inspired robotic gripper that is able to rapidly switch adhesion (in less than 0.5 s) for grasping and releasing objects by mimicking the back-scrolling behavior of the gecko toe pad. The gripper retains the fast and stable manipulation of the conventional mechanical gripper but replaces the multifinger configuration with self-peeling adhesive cantilevers that mimic the gecko toe pads. This is more reliable and has a higher load capacity than stimulus-responsive switchable adhesives (such as heat, light, voltage, magnetic field, and air pressure). In order to investigate the key parameters affecting the self-peeling behavior, the self-peeling model and the mechanism of the gripper were constructed and analyzed based on the experiment, FEA simulation, and numerical analysis, which are in great agreement. Demonstrations of fast and precise manipulation (i.e., grasping, transferring, and releasing) of materials of various sizes and weights, such as thick and thin glass substrates, fragile silicon wafers, and soft PET films, illustrate the unusual switchable adhesion capabilities of the gripper. More importantly, a strategy for active adhesion control (i.e., precise adhesion modulation) was incorporated into the gripper, providing a quantitative criterion for the gripper to adjust the adhesion strength according to the requirements of practical applications.

This robotic gripper can serve as a new end-operating gripper for a variety of applications requiring rapidly and precisely tunable adhesion, opening an avenue for the development of robotic manipulation. Future work will focus on (a) the development of grippers with a scalable size that can be applied for different manipulation conditions and (b) the manipulation of complex 3-dimensional and rough objects and surfaces by optimized grippers to enhance versatility, e.g., by integrating magnetically actuated smart adhesives proposed in our previous work or by introducing shape memory polymers [30,72,73].

## Materials and Methods

### Materials

Polydimethylsiloxane (Sylgard 184), with a 1:10 curing agent to polydimethylsiloxane base ratio, was supplied by Dow Corning. The motor (20BYGH-28mm), controller (DM420), and power supply (24 V) were supplied by Siheng Motor Manufacturing Co., Ltd, Shanghai, China. The slider, screw, guide shaft, connecting rods, hinges, and stainless steel sheet were manufactured by Chenxiao Precision Hardware & Plastic Co., Ltd, Shenzhen, China. The homemade test device was machined by Shangte 3D Technology Co., Ltd, Shenzhen, China. The silicone glue (organic silicone glue, HJ-T326) was supplied by Fangguan Industry Co. Ltd., Shenzhen, China. All materials were purchased from commercial sources and used as received, unless otherwise noted.

### Instruments

The scanning electron microscopy images were acquired by Hitachi SU8010. The adhesion data were collected using force test apparatus with a minimum accuracy of 2 mN (PT-1198GDP, PERFECT Instrument Company, Dongguan, China). The CCD system (RS-500C) for in situ observations was supplied by Ruishikeni Optical Co., Ltd, Shenzhen, China. The transfer robot (AH5005) was supplied by Quotient Kinematics Machine, Dongguan, China.

### Fabrication of the adhesion module

First, the adhesive film is fabricated by the technology of double exposure, material filling, and curing shrinkage described in our previous research [60]. Then, the adhesive film is bonded to the stainless steel sheet using silicone glue so that the 2 do not separate during the peeling process.

### Measurement and characterization of precise adhesion modulation

The 4 legs of the gripper were first pre-bent by the motion of the motor to introduce a preset peeling angle *θ_B_*. One of the legs was attached to the glass substrate with a preload force (3 N) and pulled upward in the pull-off direction until failure. This was accomplished by utilizing the force test apparatus for data exports from the personal computer. The load–pause–pull test method was used, in which the preload force was assumed to be a positive force at the contact between the leg and the glass substrate, and the pressing speed was set to 1 mm/min. The preload force was applied for 5 s to ensure full contact. Subsequently, the negative force corresponding to the adhesive force was obtained by detaching the leg and the glass substrate at a pull-up speed of 1 mm/min.

### FEA simulation of the peeling process of the adhesion module

The FEA simulation of the peeling process of the adhesion module consists of 3 parts: (a) geometric modeling, (b) boundary condition and interaction, and (c) simulation of the peeling behavior of the adhesion module. The details are provided in Note S1.

### Numerical analysis of the peeling mechanism

The numerical analysis of the peeling mechanism consists of 2 parts: (a) the superposition method for investigating the action of the peeling force *F* and the adhesion force *P_range_* on the adhesion module and (b) the energy method for solving the maximum peeling force *F_max_*. The details are provided in Note S2.

### FEA simulation of the precise adhesion modulation of the gripper

The FEA simulation of the precise adhesion modulation of the gripper consists of 2 parts: (a) geometric modeling and (b) boundary condition and interaction. The details are provided in Note S3.

## Data Availability

The data that support the findings of this study are available from the corresponding authors upon reasonable request.

## Supplementary Materials

Supplementary 1 Notes S1 to S3 Figs. S1 to S18 Tables S1 and S2 Movies S1 to S9 References [74–79]
