# Supplementary material for: Gecko Toe Pad-Inspired Robotic Gripper with Rapidly and Precisely Tunable Adhesion
Source: Research (Wash D C). 2025 Apr 23;8:0687. doi: 10.34133/research.0687 (PMC12015100; doi:10.34133/research.0687)
Supplement: Supplementary 1 — Notes S1 to S3 Figs. S1 to S18 Tables S1 and S2 Movies S1 to S9 References [74–79] [file research.0687.f1.zip › Supporting Information to Research-R1.docx]

Supporting Information

Gecko Toe Pad-Inspired Robotic Gripper with Rapidly and Precisely Tunable Adhesion

Shuai Li1,2*, Hongmiao Tian2*, Xijing Zhu1, Mingxin Liu2, Xiangmeng Li1,2, Jinyou Shao2

1Shanxi Key Laboratory of Advanced Manufacturing Technology, School of Mechanical Engineering, North University of China, Taiyuan, Shanxi 030051, China.

2Micro- and Nano-technology Research Center, State Key Laboratory for Manufacturing Systems Engineering, Xi’an Jiaotong University, Xi’an, Shaanxi 710049, China.

*Address correspondence to: Shuai Li; lishuai51@nuc.edu.cn and Hongmiao Tian; hmtian@xjtu.edu.cn

**Supplementary Note 1. FEA simulation of the peeling process of the adhesion module**

1) Geometric modeling

A peeling model based on cohesive zone theory was built using the finite element software ABAQUS/CAE 2020 to simulate the peeling behavior of the adhesion module on a smooth surface[74-76]. In order to enable cost-effective calculation, the geometric model was constructed as a two-dimensional shape due to the axial symmetry of the components, which mainly consist of stainless steel sheet, adhesive film, and glass substrate (Fig. S3A). The length *l1* and the thickness *h1* of the stainless steel sheet are 18 mm and 0.2 to 0.53 mm respectively. The length *l2* and the thickness *h2* of the adhesive film are 10 mm and 1.1 mm respectively. The length and thickness of the glass substrate are 18 mm and 1 mm respectively, with the left side of the glass substrate aligned with the left side of the adhesive module. All parameters in the simulation match the parameters of the actual structure.

2) Boundary condition and interaction

In the simulation, the bottom of the glass substrate and the right side of the stainless steel sheet were completely immobilized. The stainless steel sheet and the adhesive film were fully bonded together. The displacement-controlled load was applied upward at the top surface of the left side of the stainless steel sheet, and the direction of load application can be adjusted by customizing the user coordinate system (Fig. S3B) to simulate the different *α*. The top surface of the left side of the stainless steel sheet was coupled to a reference point, RP, and a load acting on the reference point corresponds to a load acting on the top surface of the left side. The size of the increments was automatically determined by ABAQUS.

To simulate dry adhesion and enable a cost-effective and convergent calculation, a zero-thickness cohesive surface based on the interfacial cohesive zone theory was used to replace the micropillar structures on the surface of the adhesive film for the interaction between the bottom surface of the adhesive film and the top surface of the glass substrate. Since the modulus of the adhesive film is comparatively low compared to the glass substrate, the top surface of the glass substrate was chosen as the master surface and the bottom surface of the adhesive film as the slave surface. In addition, a finite sliding contact was chosen for the definition of the contact, since the two interfaces may slide relative to each other during the peeling simulation. The cohesive behavior was described by a traction–separation response, as shown in Fig. S4. The traction *t* is defined by *P*/*A*, where *P* is the separation force at the interface during the separation process and *A* is the total area of the interface. The relationship between the traction *t* and the separation *δ* is considered as a line response, namely,

(1)

where *K* represents the interface stiffness matrix. In the simulation, the normal stiffness component *Knn* and the shear stiffness components *Kss* and *Ktt* were decoupled and all set to 8000 N/mm. The initial damage criterion of the interface adopts the quadratic nominal stress damage criterion, and its constitutive formula is[77]:

(2)

where *tn*, *ts*, *tt* are the tractions during separation and *tnmax*, *tsmax*, *ttmax* are the maximum tractions for cohesive contact. In this case, *tnmax* = *tsmax*= *ttmax* =0.4 MPa, and the fracture energy was set to 0.2 mJ. To improve the convergence of the model, the viscosity coefficient of the interface was set to 1E-5. Notably, the values of the parameters *K*, *tmax* and fracture energy were fitted from the experimental results according to Fig. 2C. Specifically, the initial pulling angle *α* in the model was first set to 90° and then the three material parameters were assigned initial values. After obtaining the simulation results, the maximum peeling force and the evolution of the peeling force were compared with the experimental results according to Figure 2C. Subsequently, the values of the three material parameters were adjusted to make the simulation results match the experimental results as closely as possible. Finally, the obtained optimal material parameters were applied to the material parameters of models with different initial pulling angles.

3) Simulation of peeling behavior of the adhesion module

In the numerical simulation, the material constants of the stainless steel sheet were set to the following values using the linear-elastic model: *E* = 190 GPa, *v* = 0.305. The hyperelastic Mooney–Rivlin model with the material constants C10 = 0.201 MPa, C01=0.041 MPa, D1 = 0.0019 was selected for the material behavior of the adhesive film. The linear elastic model with the material constants *E* = 55 GPa, *v* = 0.25 was selected for the material behavior of the glass substrate. To systematically analyze the simulation results, the reaction force, the energy and the scalar stiffness degradation for cohesive surfaces (CSDMG, which is used to determine the failure of the adhesive interface) were set in the history output variables (Fig. 2E and Figs. S5 and S7). The maximum normal stress of the interface can be extracted from the stress distribution of each incremental step on the adhesive interface (Fig. S6). The relevant material constants are listed in Table S1.

**Supplementary Note 2. Numerical analysis of the peeling mechanism**

1) Superposition method for investigating the action of the peeling force *F* and the adhesion force *Prange* on the adhesion module

The effect of *M*(*F*) on the deflection and angle of rotation of the adhesion module is first calculated as shown in Fig. S11A, where the axial displacement caused by *Fx* is neglected and *α*>>*θB*, resulting in:

(3)

where *a* is the length of the end of the stainless steel sheet, *d* is the component of *a* in the direction perpendicular to *F*, and . *lcrack* is the crack variation during the stable stage and *lcrack* < *l* (the length of the adhesive film). The boundary condition is as follows:

(4)

where Equation S4 means that there is no variation in deflection and angle of rotation at the initial crack point C, i.e., point C is the origin of the *x*-axis.

Substituting Equation S3 into and applying Equation S4 gives the angle of rotation and deflection of the adhesion module at any point on the *x*-axis under the action of the peeling force *F*:

(5)

(6)

Substitute the *x*-coordinate at point B, i.e., *lcrack*, into the two equations above to obtain the angle of rotation and the deflection at point B under the action of the peeling force *F*:

(7)

(8)

Next, the effect of *M*(*Prange*) on the deflection and angle of rotation of the adhesion module is calculated as shown in Fig. S11B, where *Prange* is approximated as a uniform load and taken from the experimental result (~150 kPa) and its region of action is *lcrack*, which gives:

(9)

where and , *b* is the width of the adhesion module, and Equation S9 becomes:

(10)

Substituting Equation S10 into and applying Equation S4 gives the angle of rotation and deflection of the adhesion module at any point on the *x*-axis under the action of the adhesion force *Prange*:

(11)

(12)

Substituting the *x* coordinate at point B, i.e., *lcrack*, into the above two equations gives the angle of rotation and deflection at point B under the action of the adhesion force *Prange*:

(13)

(14)

According to the principle of the superposition method, by superimposing the angle of rotation and the deflection at point B under the action of *F* and *Prange*, respectively, the total angle of rotation and the total deflection at point B can be obtained as follows:

(15)

(16)

2) Energy method for solving the maximum peeling force *Fmax*

The work *WF* done by the peeling force *F* consists of two components (neglecting axial displacement), namely, the work done by *Fy* to deform point B by *ωB* deflection and the work done by *F*·*d* moment to deform point B by *θB* angle of rotation. The expression for *WF* is as follows:

(17)

The elastic strain energy *Uε* stored in the adhesion module during the stable stage also consists of two components, namely,

(18)

where *Ub,g* and *Us,t* represent the bending strain energy stored in the stainless steel sheet and the tensile strain energy stored in the adhesive film, respectively. According to the mechanics of materials[64], *Ub,g* is expressed as:

(19)

where this portion of the energy is not dissipated during the peeling process due to the high elastic modulus of the stainless steel sheet.

For *Us,t*, it exists in the BC range, i.e., in the *lcrack* range. The deformation *ωgf* of the adhesive film in the BC range is the difference between the deflections derived from Equations S6 and S12, i.e.,

(20)

Since the material used for the adhesive film is an isotropic, hyperelastic material and the deformation is in the linear region of the stress–strain curve, the expression for *Us,t* is:

(21)

where this portion of the energy is dissipated during the peeling process due to the low elastic modulus of the adhesive film.

The free energy *UГ* is related to the thermodynamic work of adhesion, *Wad*, of the adhesive film, which is expressed as follows[6, 66]:

(22)

**Supplementary Note 3. FEA simulation of the precise adhesion modulation of the gripper**

1) Geometric modeling

This FEA simulation consists of two processes, i.e., the pre-bending of the adhesion module and the attachment/detachment from the glass substrate. The geometric model of the pre-bending process is the same as that without glass substrate in Supplementary Note 1, but has a thinner adhesive film (the thickness *h3* is 0.5 mm and is consistent with that of the actual structure), as shown in Fig. S15A. After pre-bending, the deformed adhesion module was saved as a new part and imported into a new model. The part of the glass substrate with the same parameters as in Supplementary Note 1 was also built in the new model. The glass substrate and the deformed adhesion module constitute the geometric model of the attachment/detachment process, as shown in Fig. S15B.

2) Boundary condition and interaction

In the simulation of pre-bending, the right side of the stainless steel sheet was completely immobilized. The direction of load application was set at 39.53° to simulate *α* = 39.53°. Both the bending force and the external energy increase with increasing *θB* due to the increasing deformation of the adhesion module, as shown in Fig. S16.

In the simulation of attachment/detachment, the bottom surface of the glass substrate was fully fixed. The displacement-controlled load was applied to the entire top surface of the stainless steel sheet in the *y*-direction, as shown in Fig. S15B. The entire top surface of the stainless steel sheet was coupled to a reference point, RP, and a load applied to the reference point corresponds to a load applied to the entire top surface. The other information not mentioned corresponds to that in Supplementary Note 1.


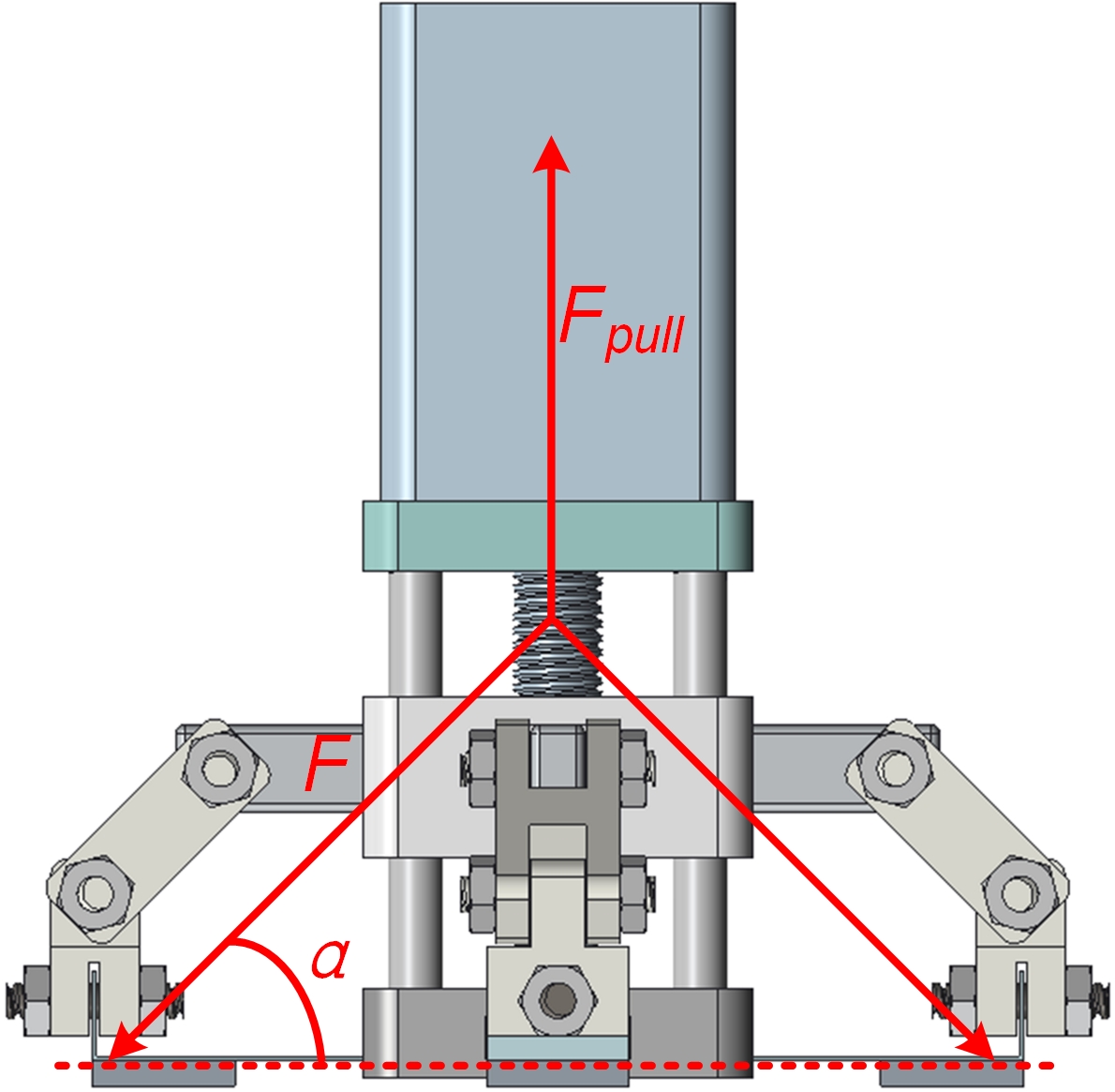


**Fig. S1.** Mechanical model of the gripper. The relationship between the pulling force of the motor, *Fpull*, and the peeling force, *F*, and the initial pulling angle, *α*, is .


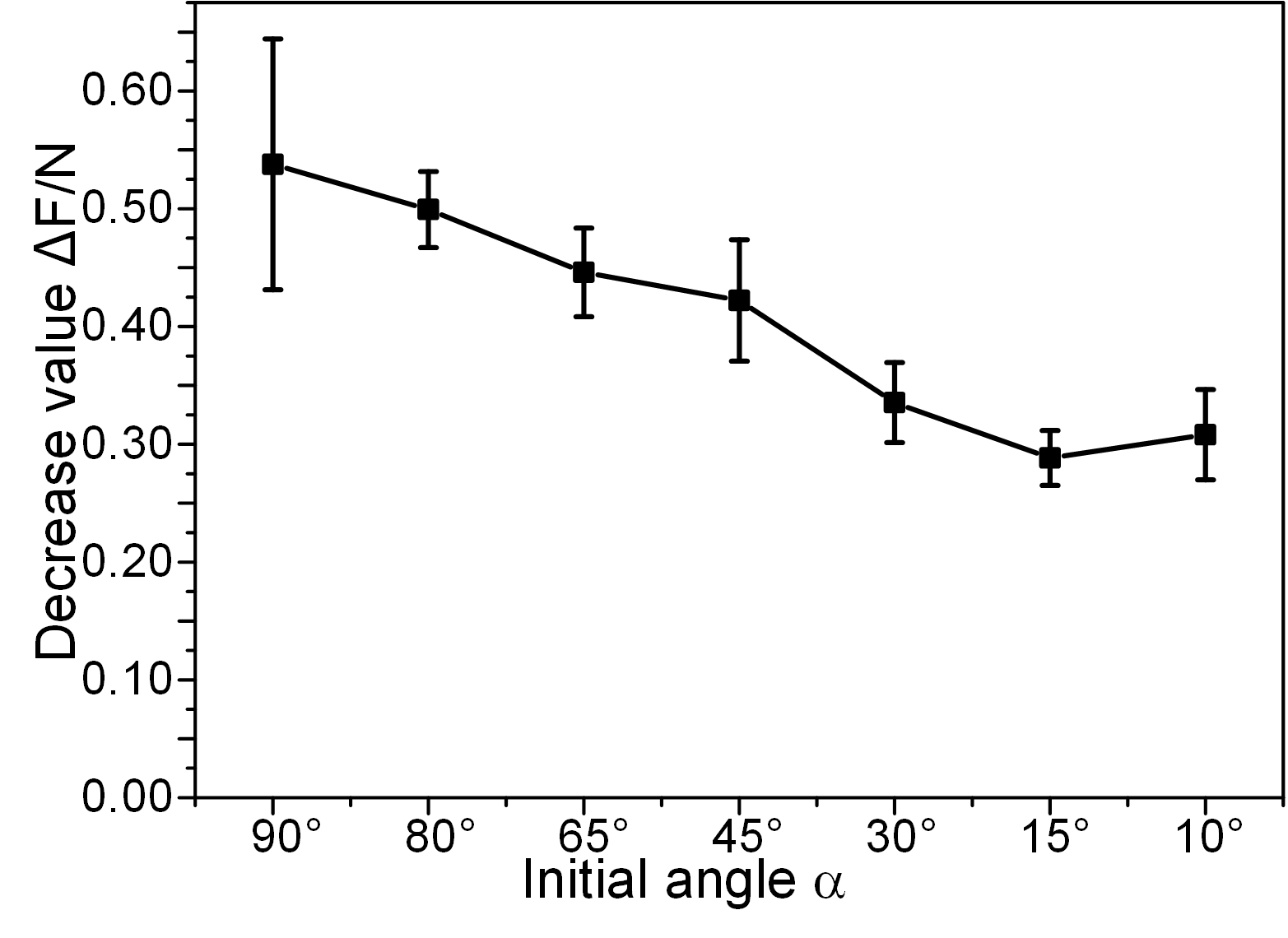


**Fig. S2.** Decreasing value *∆F* of the peeling force from Stage I to Stage II in Fig. 2C. As the initial pulling angle *α* decreases, the value *∆F* tends to decrease and increases after 15°. This means that the ratio between the elastic strain energy of the adhesion This means that the ratio of the elastic strain energy of the adhesion module and the external work performed by the force test apparatus gradually increases as *α* changes from 90° to 15°, while the ratio between the free energy required to create a new surface and the external work performed by the force test apparatus decreases and reverses slightly after 15°. Error bars represent the SD for n = 3


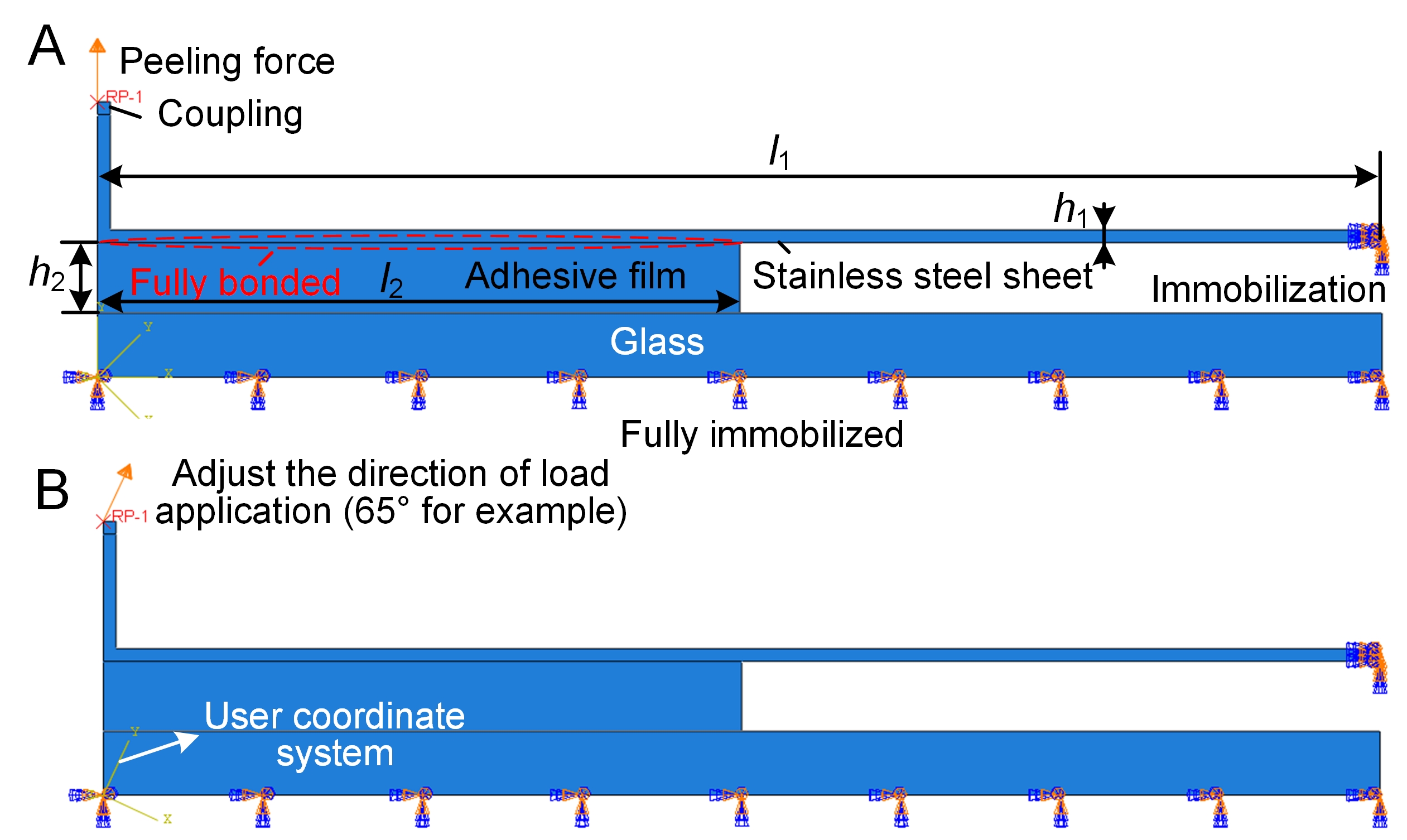


**Fig. S3.** Schematic diagram of the geometric model of the finite element simulation. (A) The geometric model was built as a two-dimensional shape mainly consisting of stainless steel sheet, adhesive film and glass substrate. (B) The direction of load application can be adjusted by customizing the user coordinate system to simulate the different *α*.


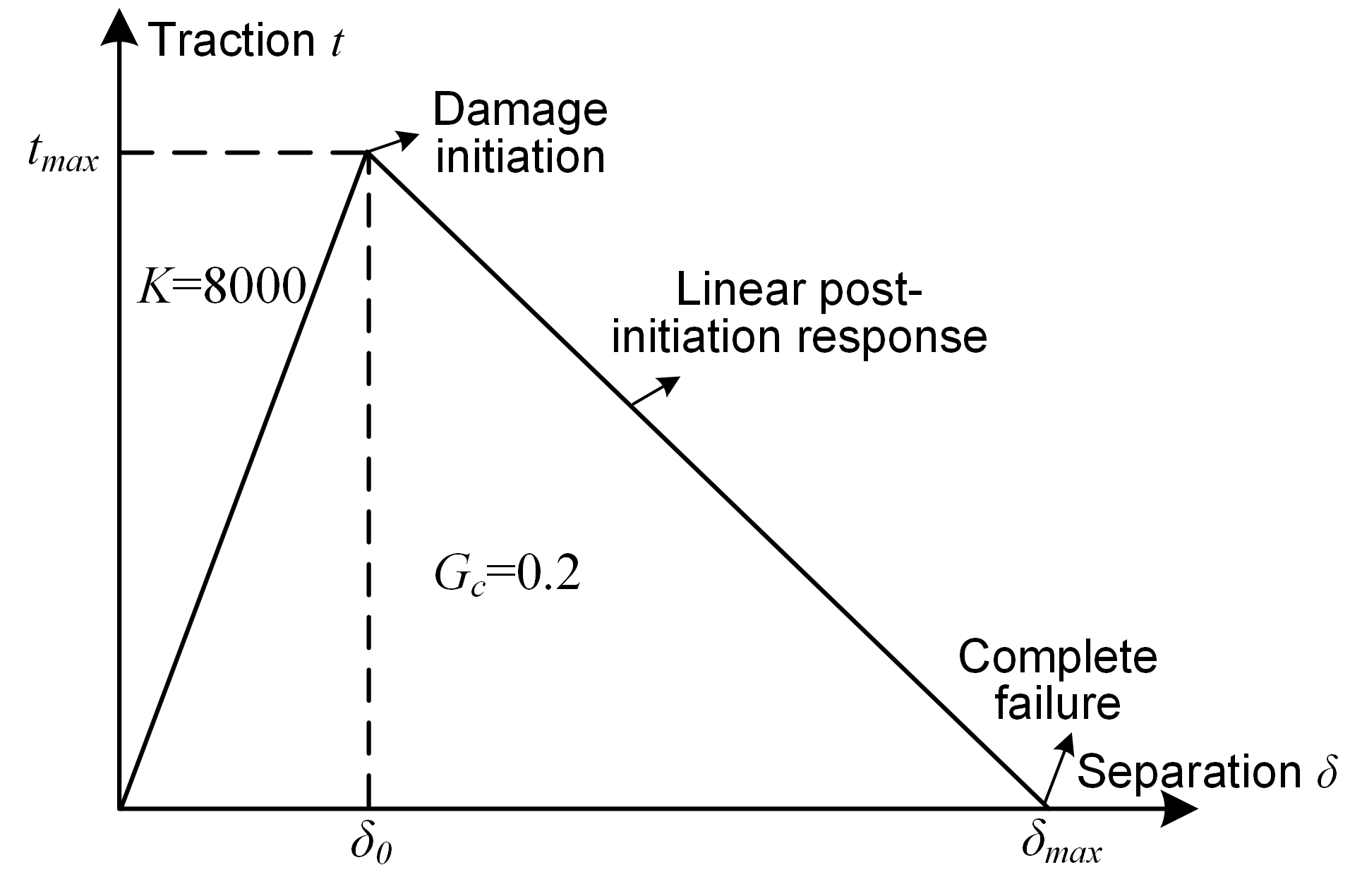


**Fig. S4.** Sketch of the traction–separation response for cohesive behavior of adhesion module.


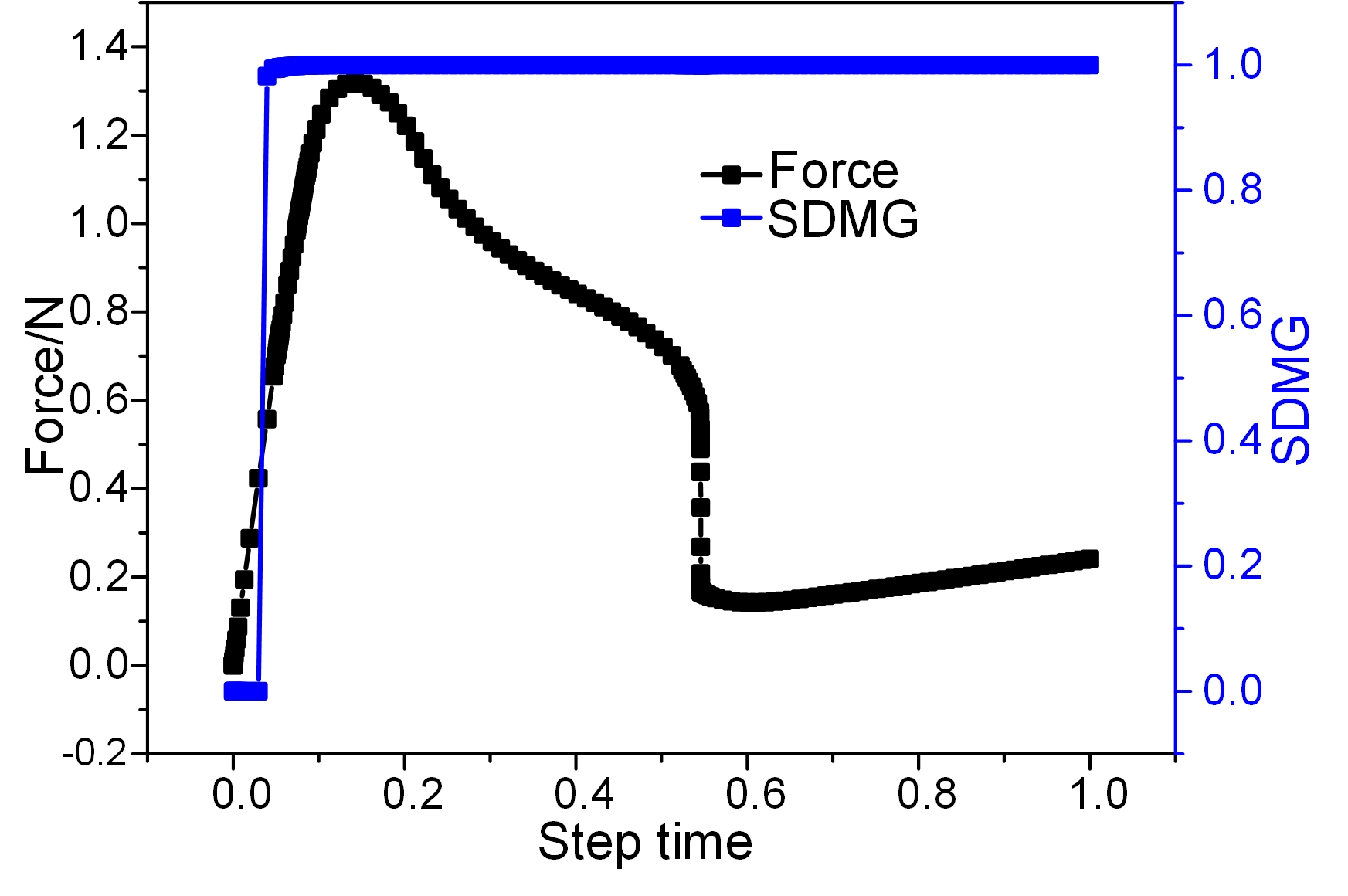


**Fig. S5.** Variation curve of the CSDMG of the first damage node of the adhesive film as a function of the step time. A CSDMG value of 1 for a node means that the adhesion of the node has completely failed. The figure shows that when the adhesion of the first damaged node of the adhesive film fails completely, the *Fmax* is only reached after a certain step time. This means that *Fmax* is reached at the time when the initial crack *lcrack0* is generated, which is consistent with the experiment and with Griffith's energy criterion for fracture.


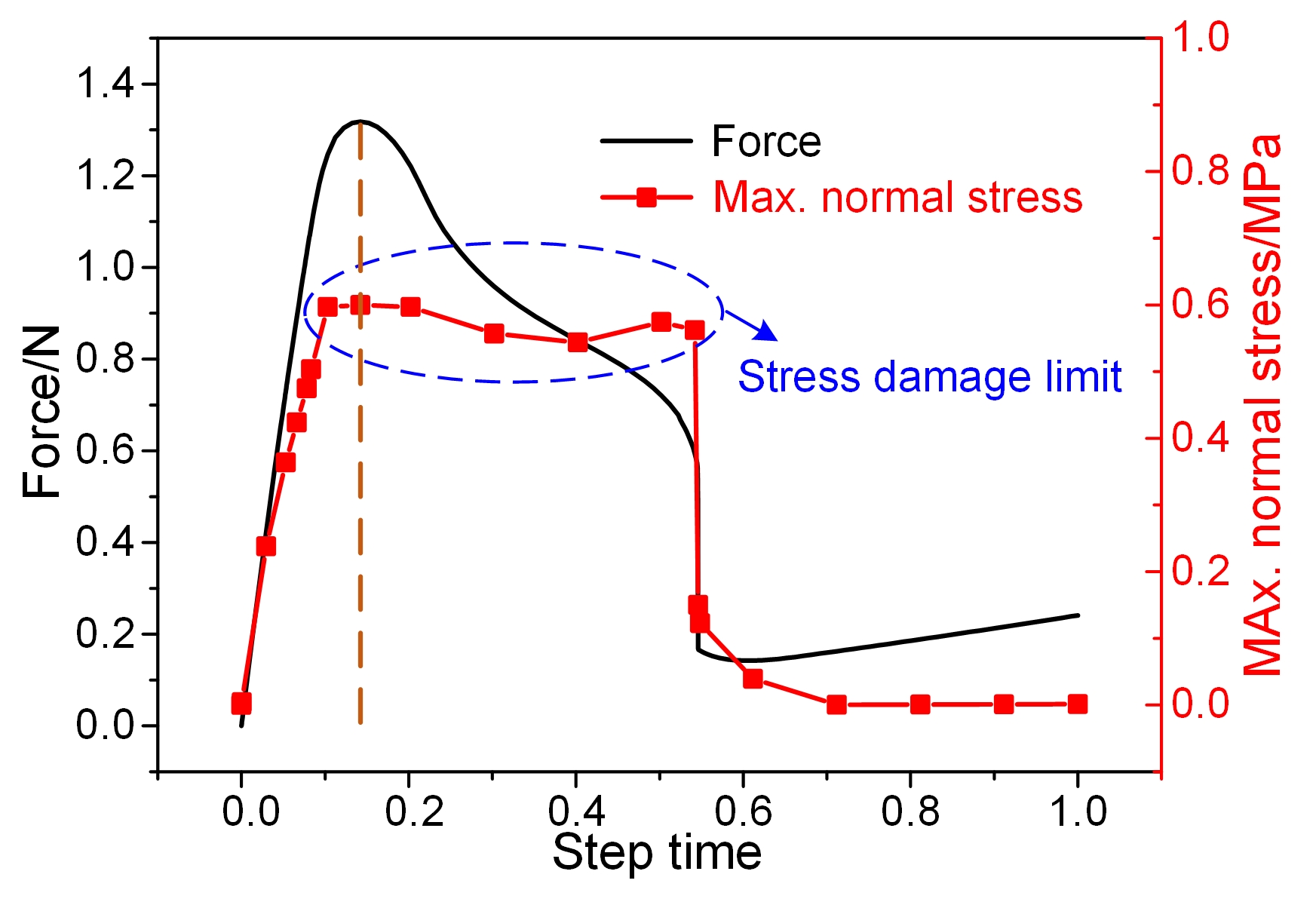


**Fig. S6.** Evolution of the maximum normal stress of the adhesive film during the peeling process. This is another logical explanation for the initiation and propagation of cracks at the interface. The first damaged node of the adhesive film experiences an increase in normal stress with increasing peeling force until the stress damage limit is reached, which means that the adhesion of the node has completely failed. At this point, however, the *Fmax* has not yet been reached, as indicated by the fact that the maximum normal stress of the first damaged node is to the left of the brown line. When the normal stresses of the next few nodes (these nodes form the initial crack *lcrack0*) reach the stress damage limit, the *Fmax* is reached, which confirms the conclusion in Figs. 2D and 2F that the *Fmax* occurs at the time of initial crack formation. Subsequently, the interfacial adhesion gradually fails as the subsequent nodes reach the stress damage limit one after another, and the crack propagation occurs until the adhesive film is completely peeled off the glass substrate (the moment when the maximum normal stress and the peeling force rapidly drop). It should be noted that the stress damage limit in the figure is a range rather than a critical value, since the criterion for interface fracture damage in the interaction module of ABAQUS is set as a quadratic nominal stress damage criterion.


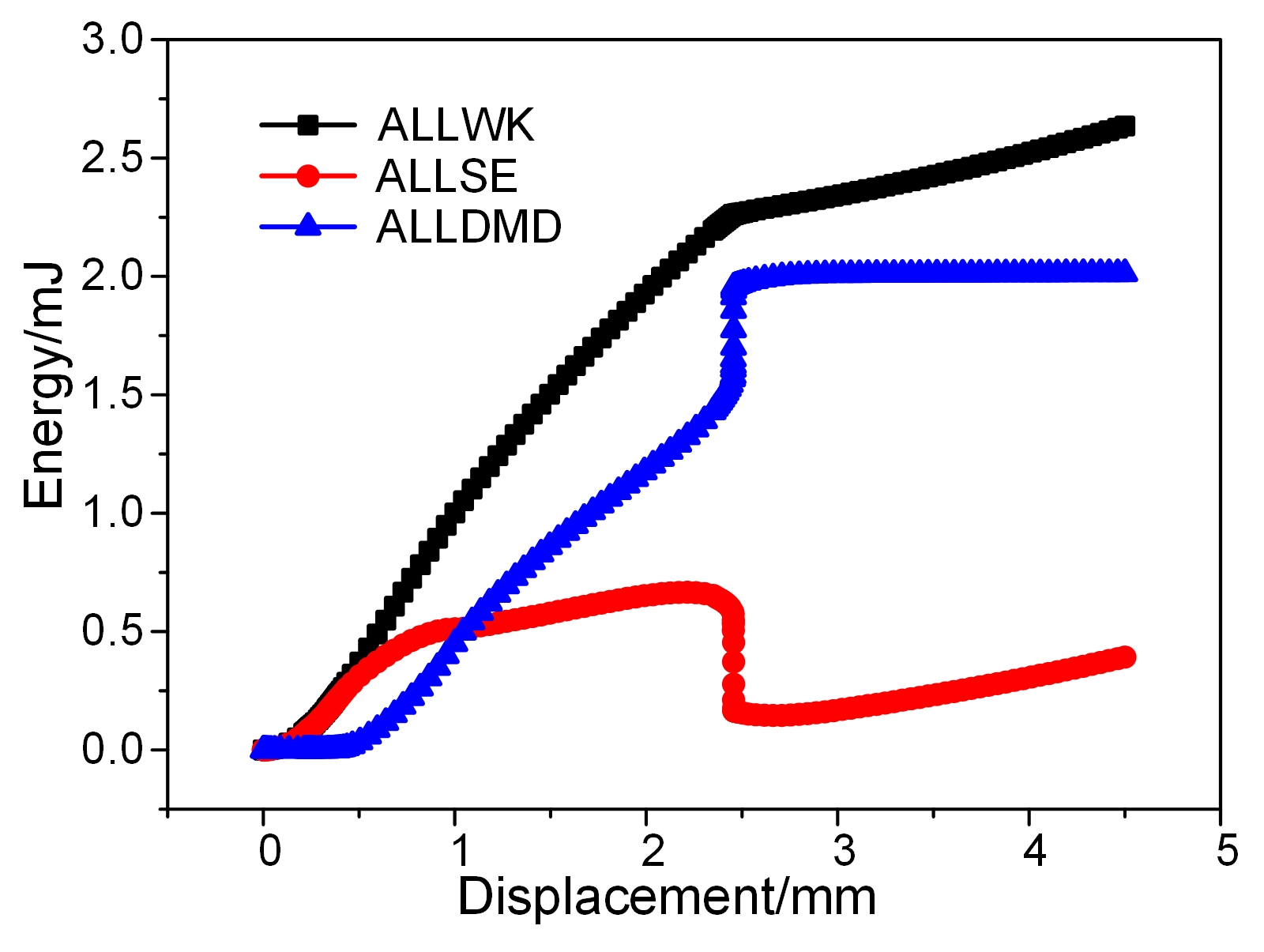


**Fig. S7.** Evolution of the energy of each component as a function of the displacement in the peeling simulation. The external work, strain energy and damage dissipation energy are denoted in ABAQUS as ALLWK, ALLSE and ALLDMD respectively. The relationship between them is ALLWK>ALLSE+ALLDMD, and the difference includes the dissipation energies ALLVD for viscosity, ALLFD for friction, and ALLCD for viscoelasticity, among others, which are not discussed due to their small values. The figure shows that the energy of ALLWK and ALLSE increases gradually with the application of the displacement load before the adhesive film is completely detached, while the energy of ALLDMD increases gradually with the application of the displacement load only after crack generation. When the adhesive film peels off completely, ALLWK continues to increase, but at a slower rate, as the external force is only doing work on the deformation of the stainless steel sheet during this period; ALLSE experiences a sharp drop before increasing again, which is due to the stainless steel sheet continuing to store strain energy after the elastic strain energy of the adhesive film has been released instantaneously. In addition, the variation pattern of ALLSE should be similar to the trend of peeling force, and indeed this is the case (Fig. 2E); ALLDMD increases sharply and then reaches a stable value, indicating that crack propagation is complete and no further energy dissipation is required.


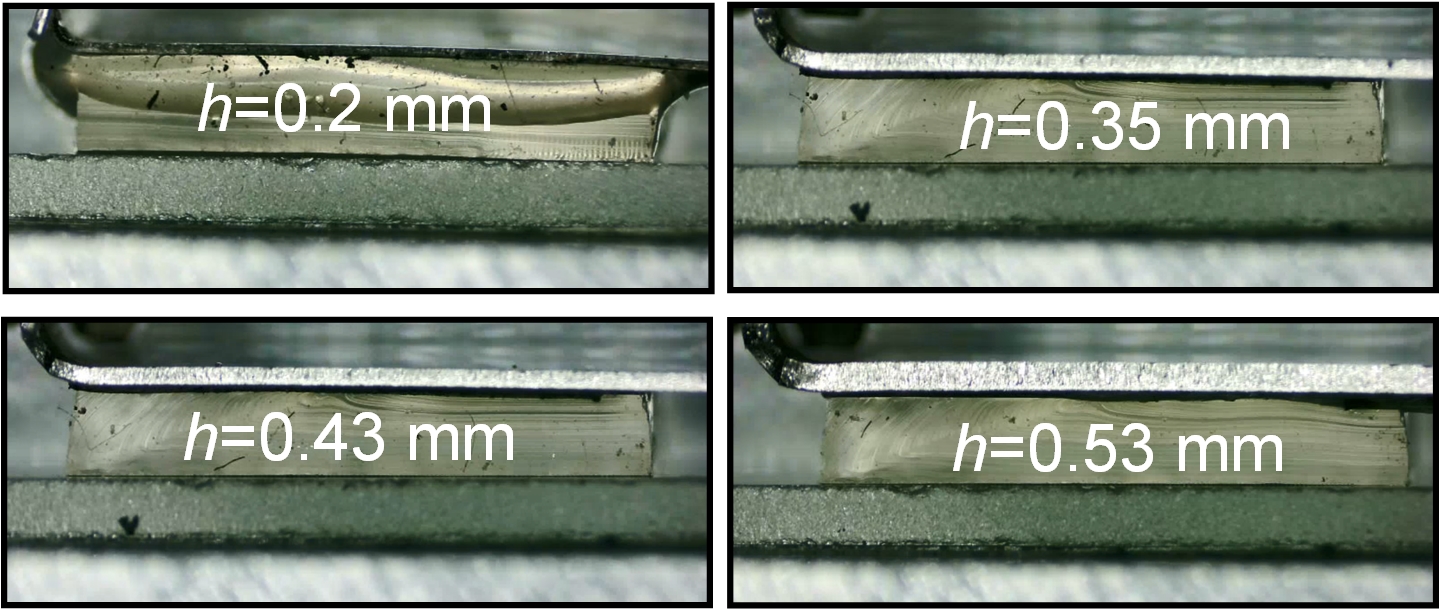


**Fig. S8.** Optical diagrams of stainless steel sheets with different thicknesses *h*.


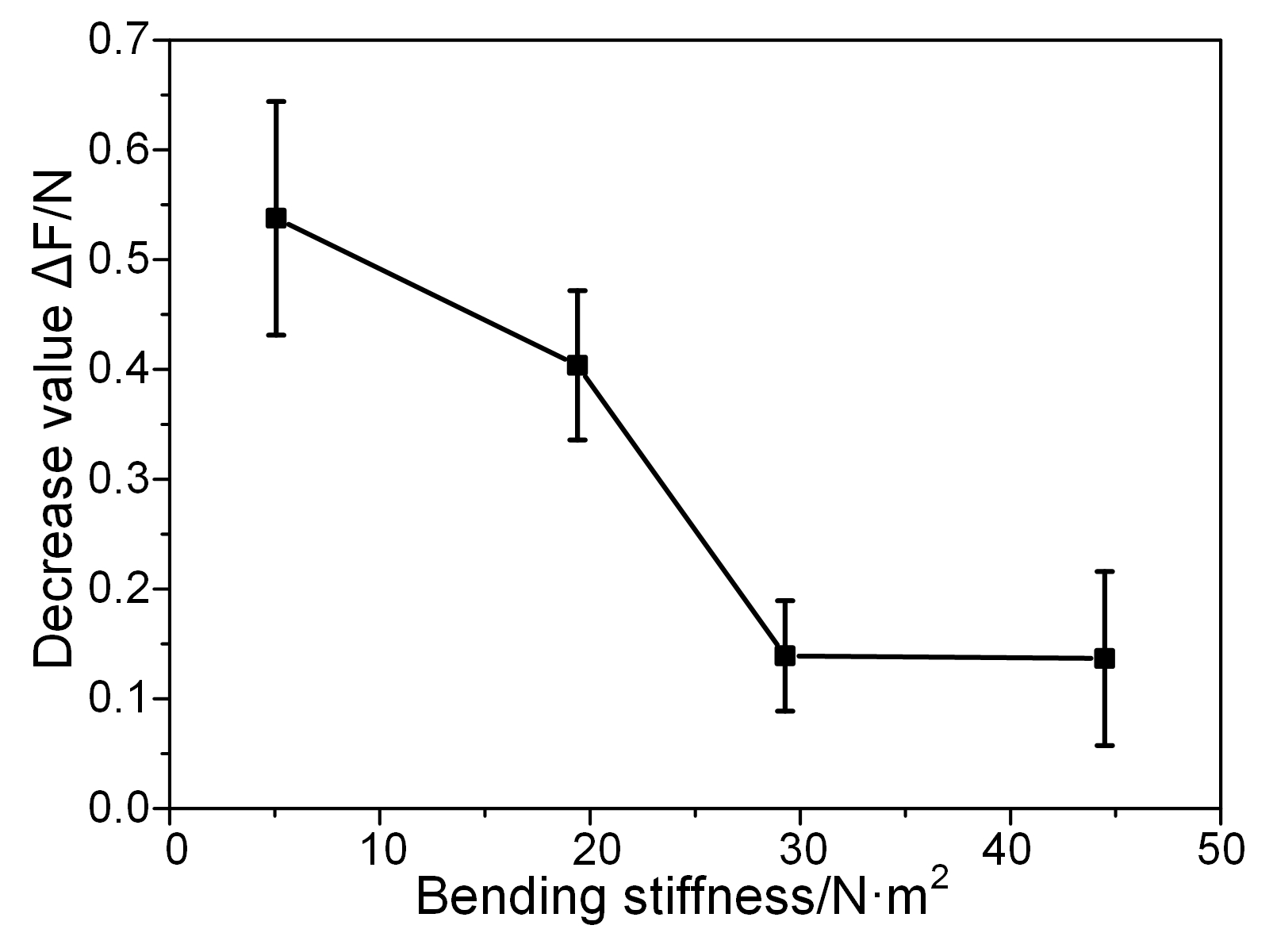


**Fig. S9.** Decrease value *∆F* of the peeling force in Fig. 2G. As the bending stiffness *EI* increases, the *∆F* tends to decrease. This means that the ratio between the elastic strain energy of the adhesion module and the external work performed by the force test apparatus increases as *EI* increases, while the ratio between the free energy required to create a new surface and the external work performed by the force test apparatus decreases. Error bars represent the SD for n = 3


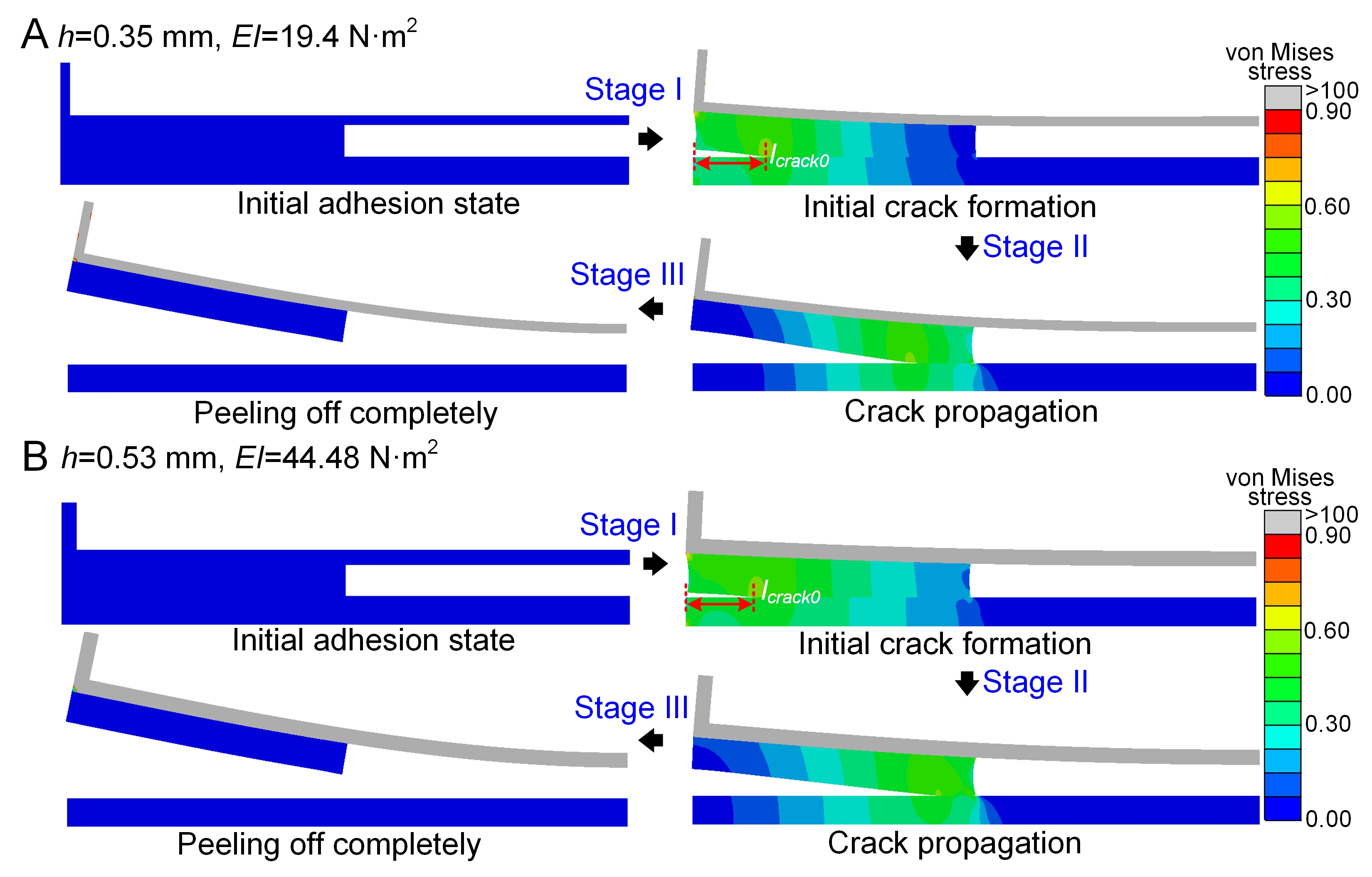


**Fig. S10.** Peeling models for different *EI*, with the cloud atlas representing the von Mises stress distribution. Using *EI* = 19.4 N·m2 and *EI* = 44.48 N·m2 as examples, the peeling models only change the thickness of the stainless steel sheet in the Part module of ABAQUS and keep the other parameters constant.


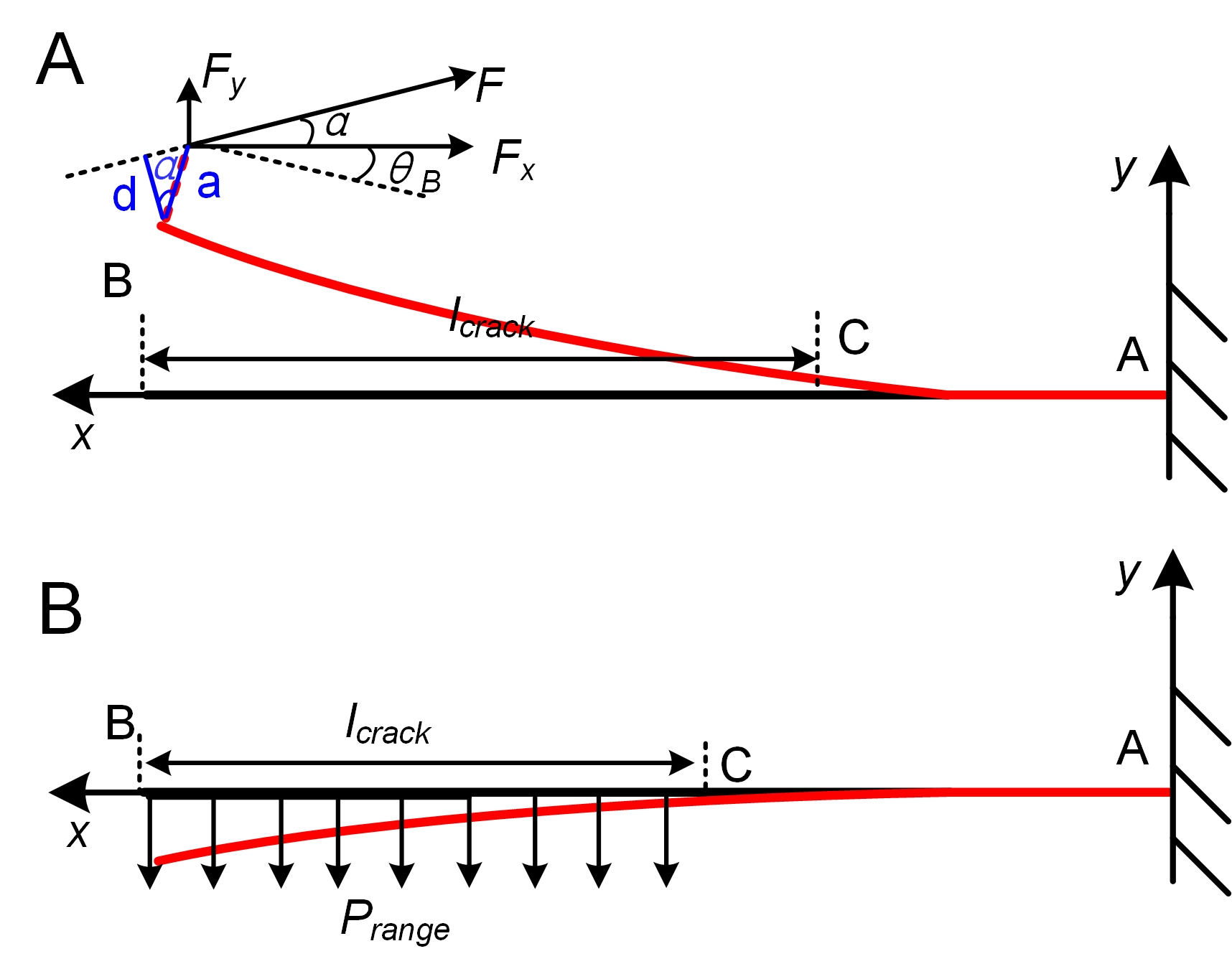


**Fig. S11.** Decoupling the action of the peeling force *F* and the adhesion force *Prange* on the adhesion module. **(**A) Action of the peeling force *F* on the adhesion module. (B) Action of the adhesion force *Prange* on the adhesion module.


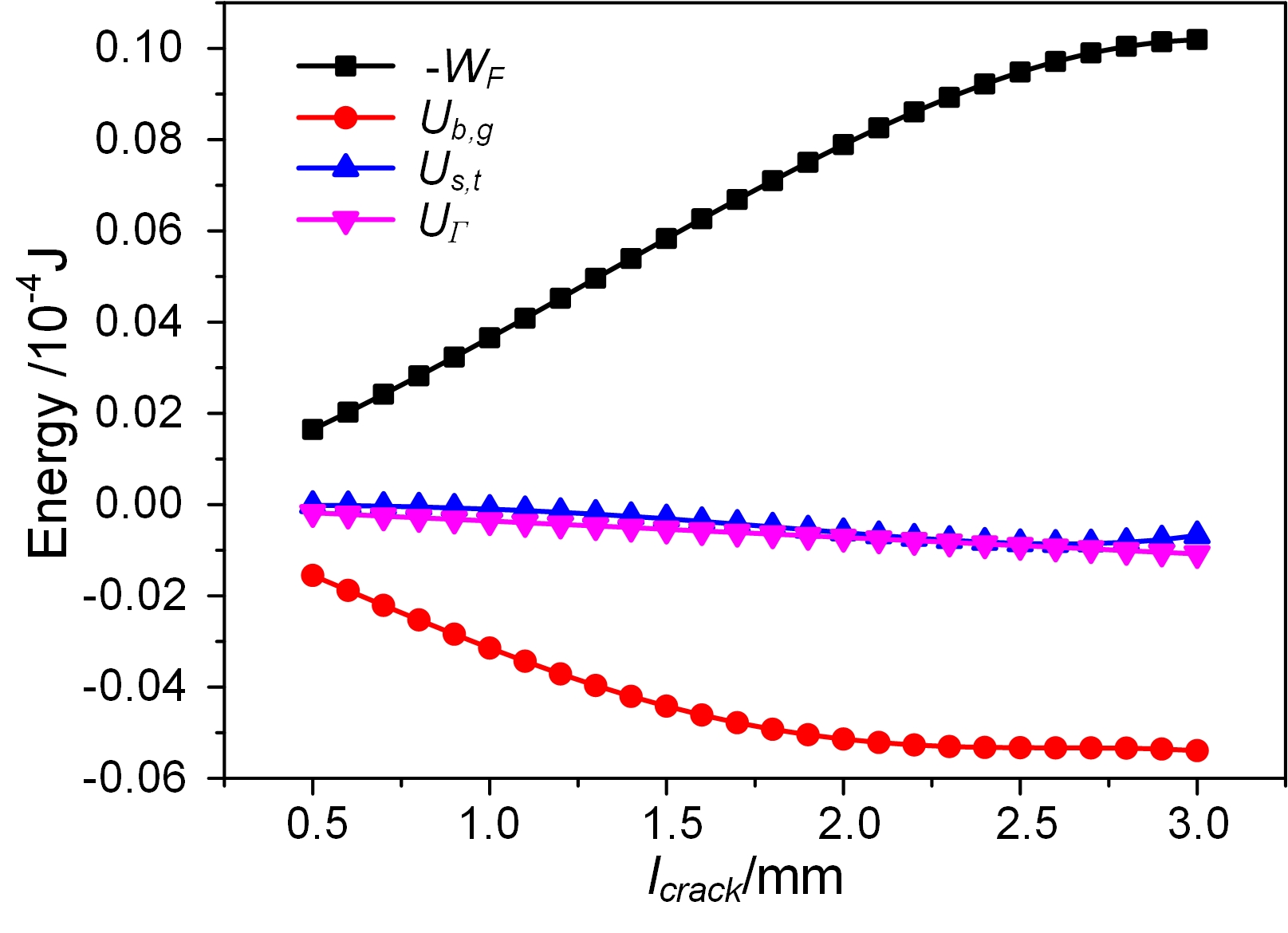


**Fig. S12.** Evolution of the energy of each component with *lcrack* during the stable stage.With increasing *lcrack*, *WF* gradually increases, and *Ub,g*, *Us,t* and *UГ* also gradually increase and have opposite signs to *WF*, indicating that *WF* is converted into the latter three components and *Ub,g* accounts for most of the energy conversion.


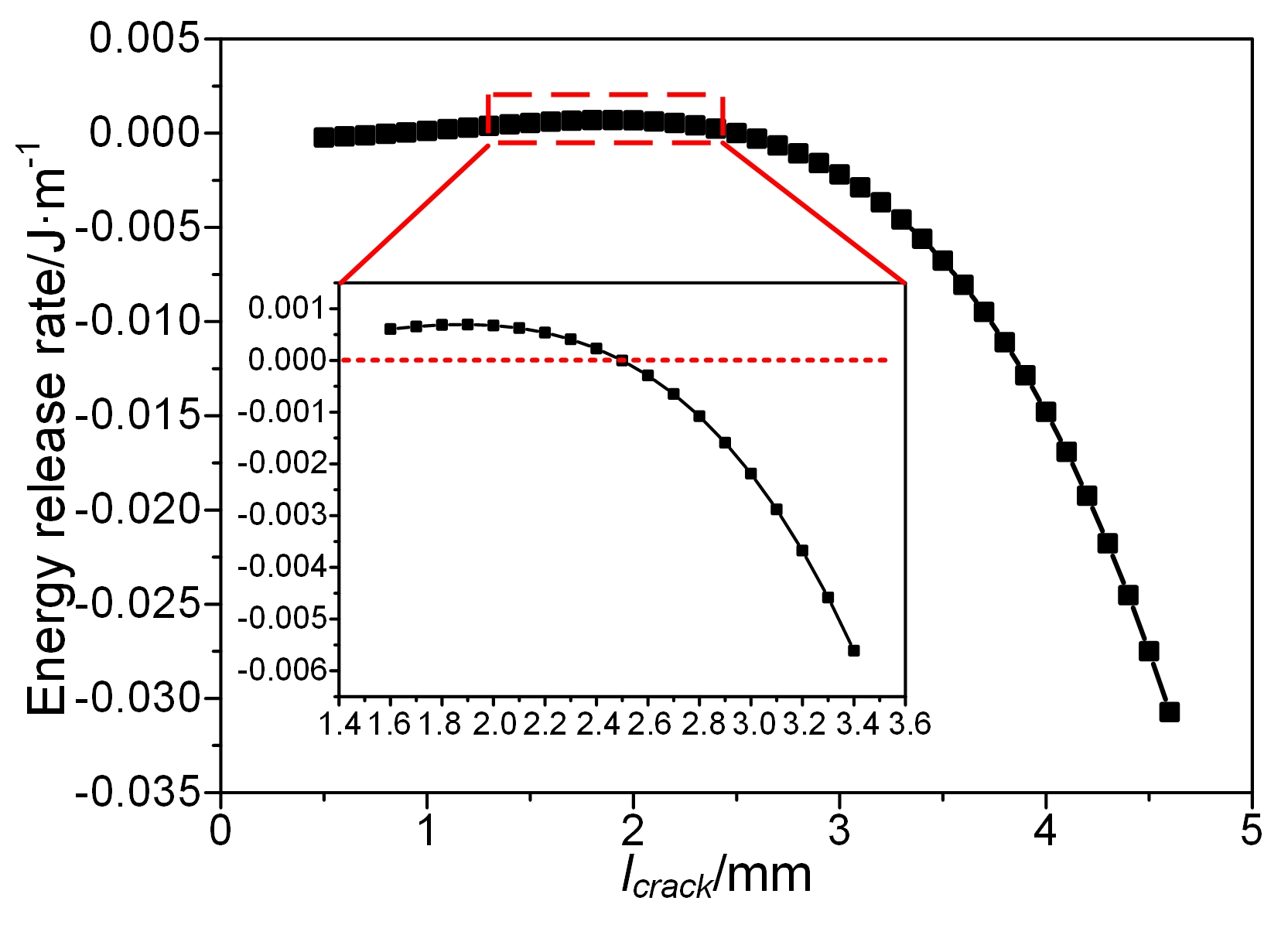


**Fig. S13.** Evolution of the energy release rate in the crack region (0.5–4.5 mm) near the initial crack *lcrack0*. The inset shows a local enlargement of the curve in the red dashed box. The figure shows that the energy release rate is positive when *lcrack* < *lcrack0* and that the energy release rate is negative when *lcrack* > *lcrack0*.


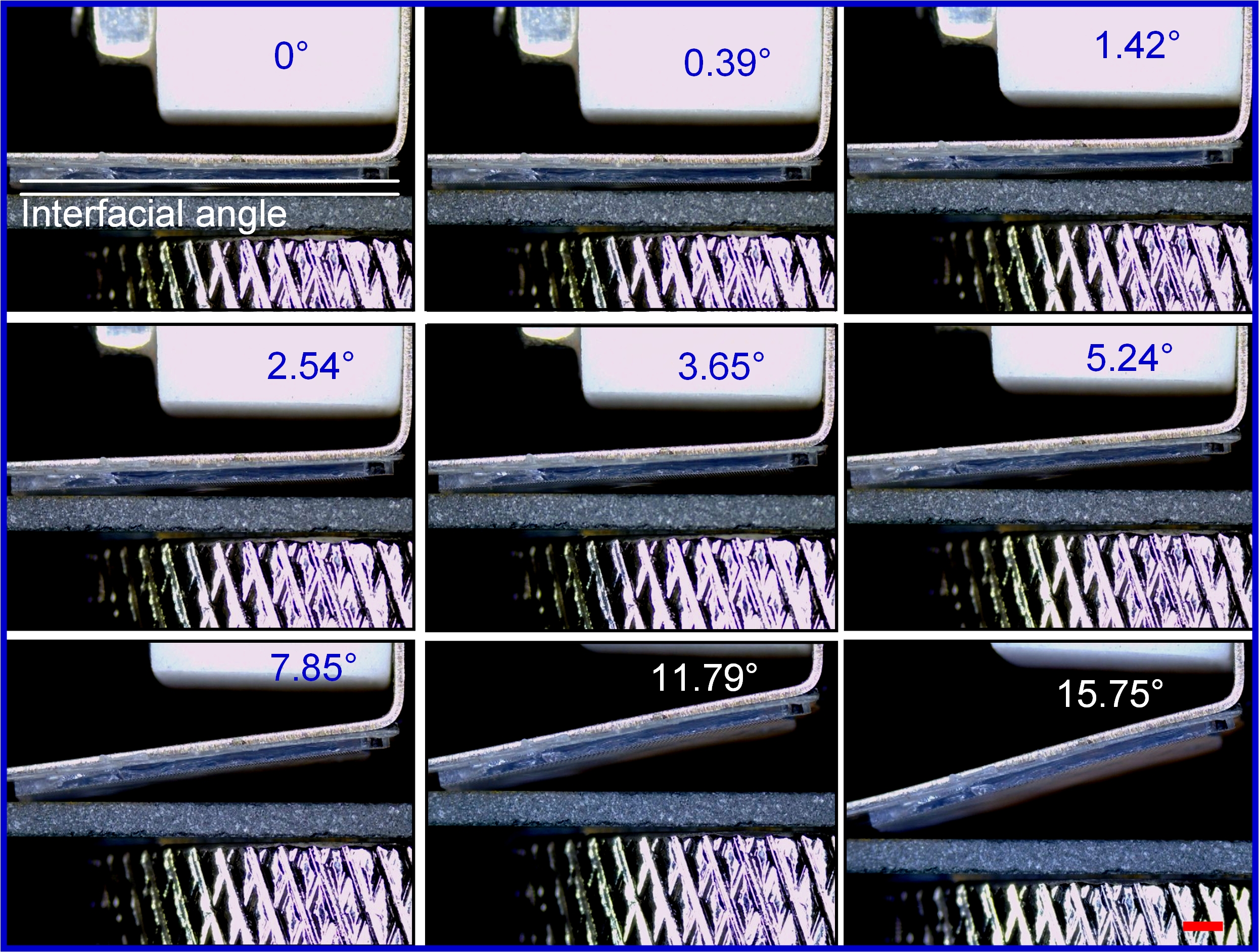


**Fig. S14.** Various preset peeling angles *θB* (from 0° to 15.75°) introduced by the motion of the motor. The scale bar corresponds to 1 mm.


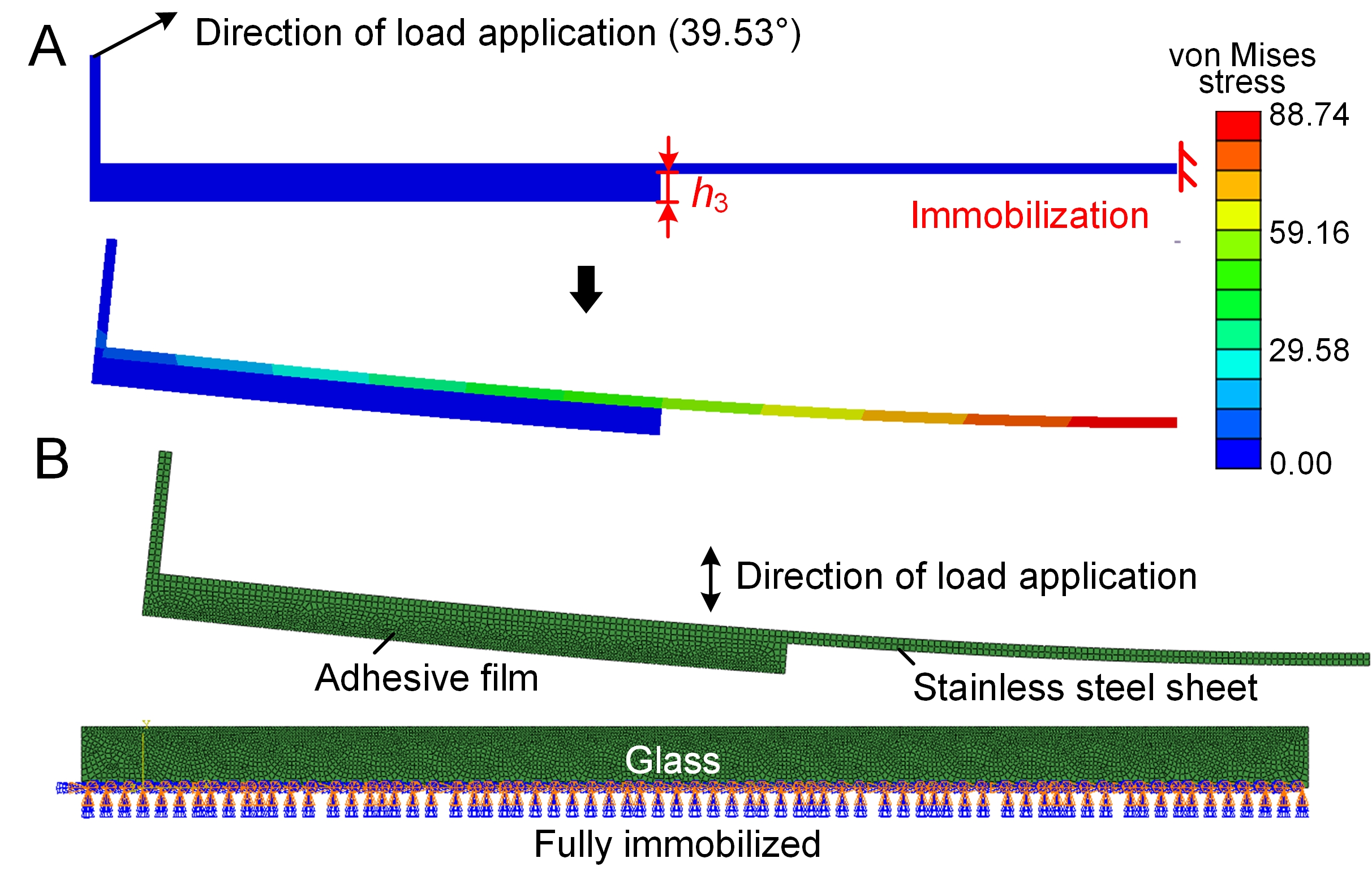


**Fig. S15.** Simulation model of precise adhesion modulation. (A) Pre-bending process of the adhesion module, with the cloud atlas representing the von Mises stress distribution. (B) Boundary condition and interaction in the attachment/detachment process.


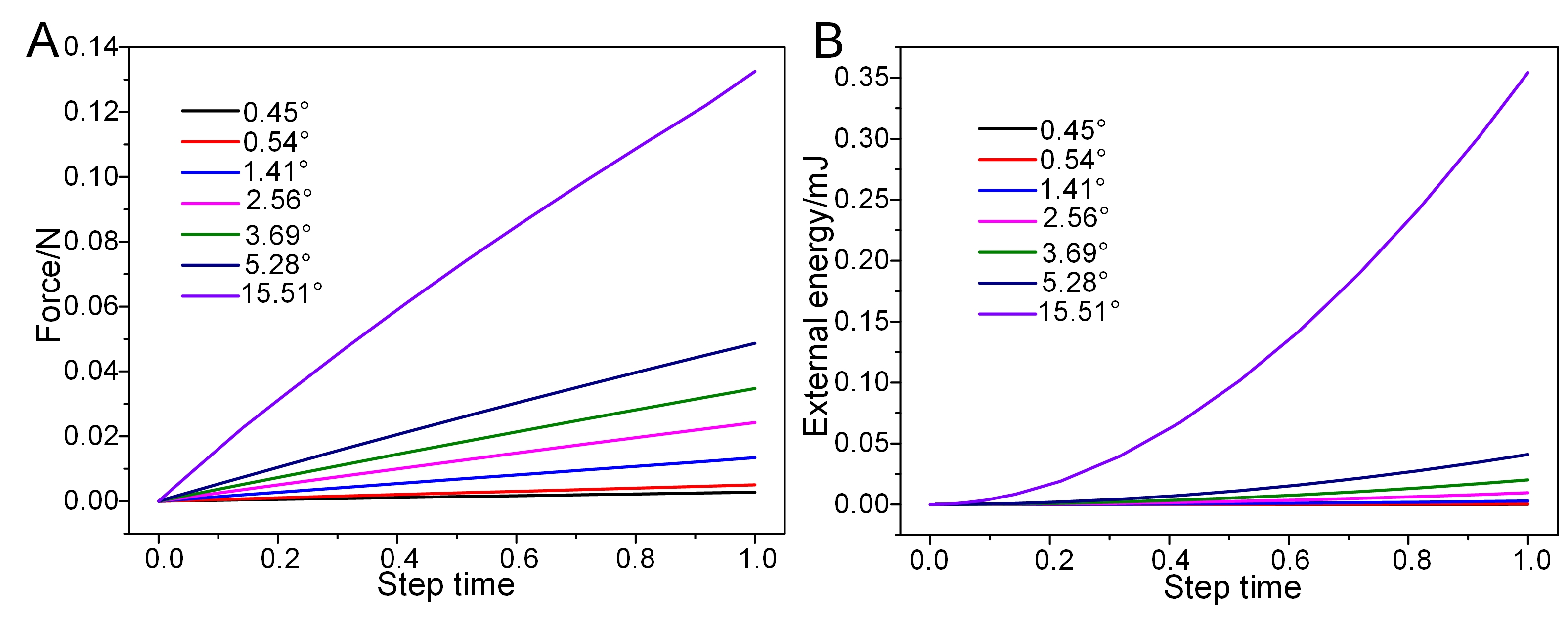


**Fig. S16.** Simulation results of the pre-bending process. (A) Variation curves of bending force versus step time at different *θB*. (B) Variation curves of external energy versus step time at different *θB*.


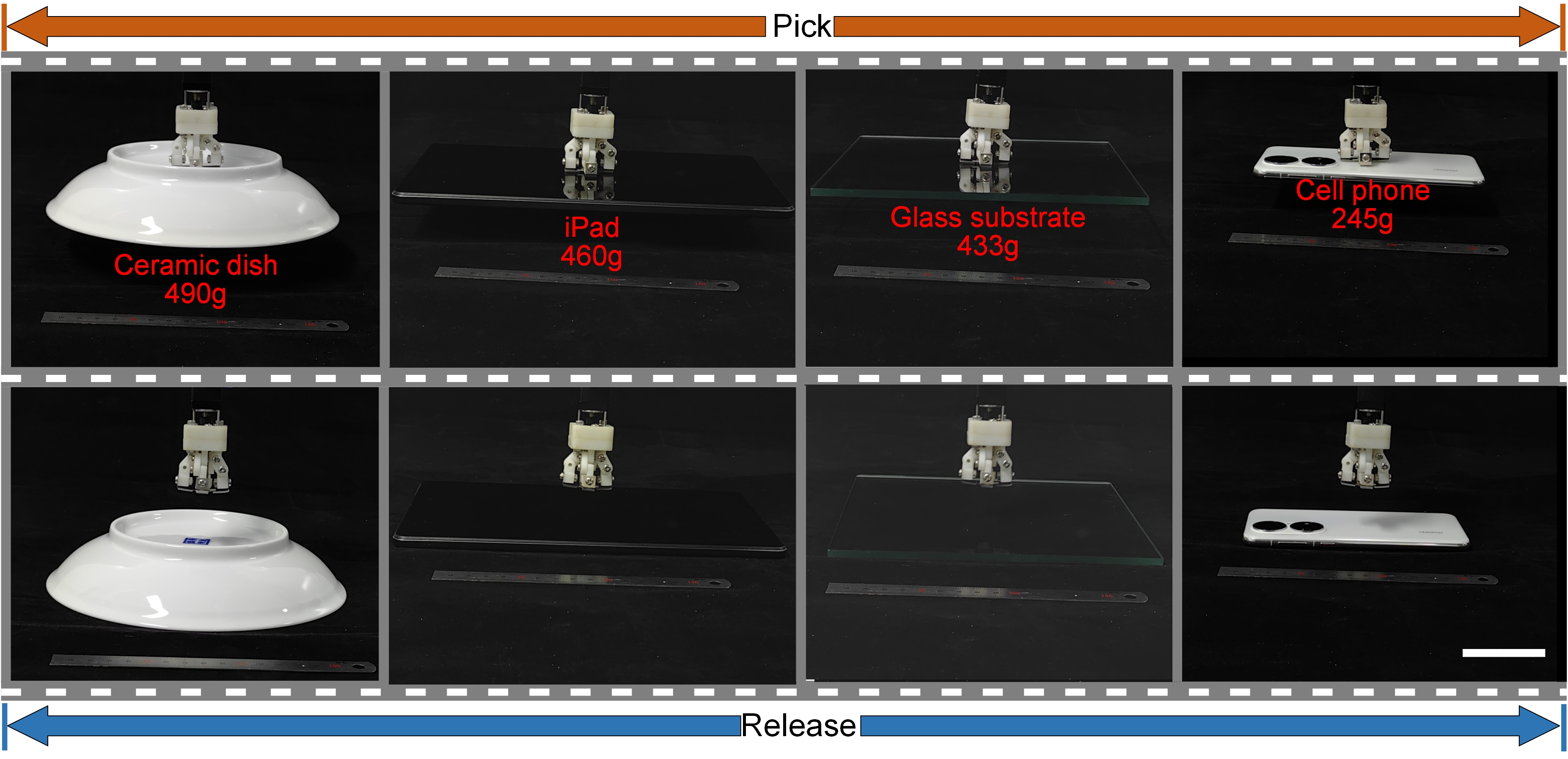


**Fig. S17.** Demonstration of the gripper handling relatively rigid and large objects, such as a ceramic dish (490 g), iPad (460 g), glass substrate (433 g), and cell phone (245 g). The scale bar corresponds to 5 cm.


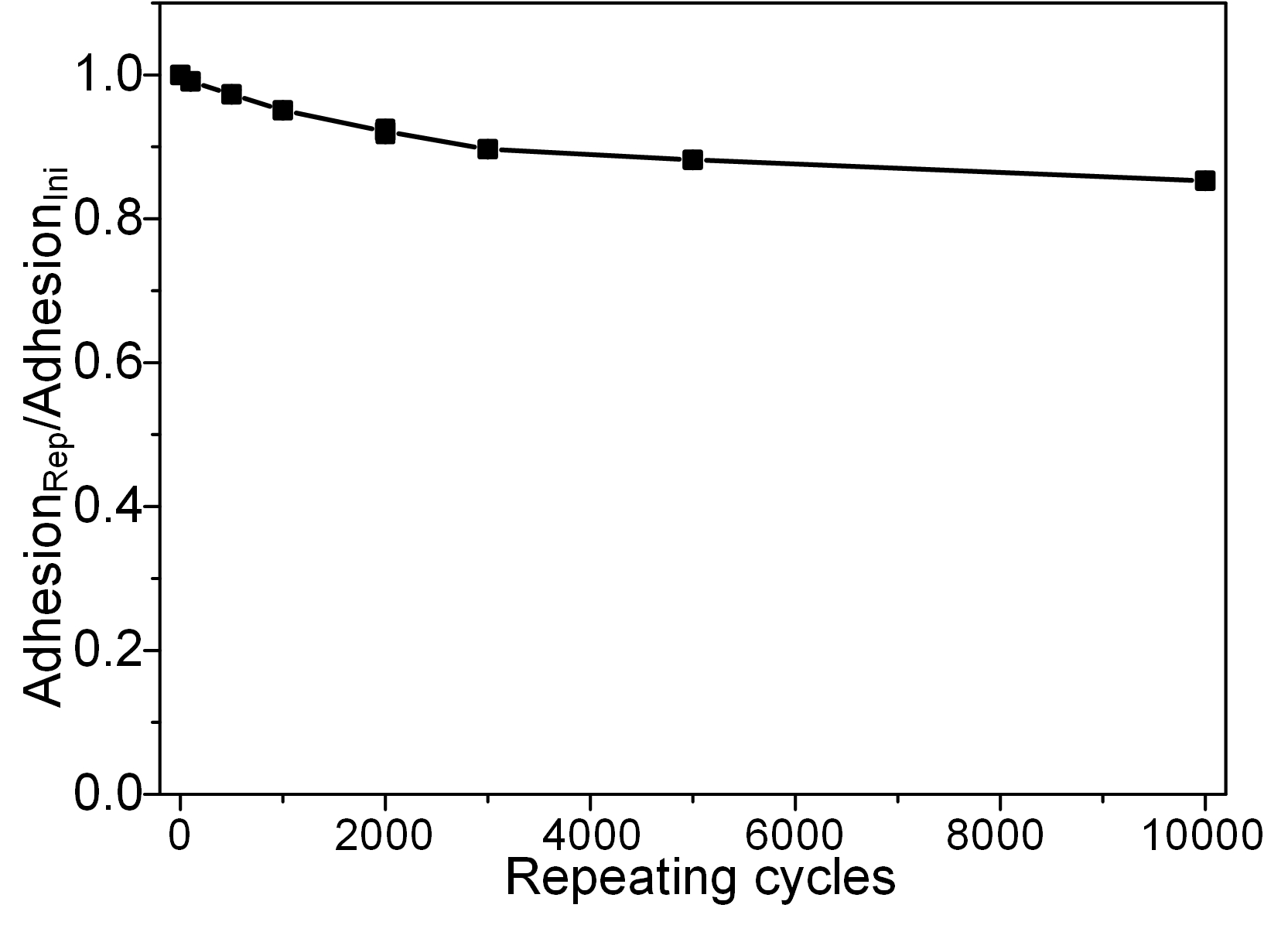


**Fig. S18.** Reusability of the gripper.After 10,000 cycles of manipulating commercial acrylic panels, the adhesive force of the gripper decreases by 14.7% and still exhibits a high value, demonstrating the robustness of the gripper. AdhesionIni and AdhesionRep stand for the initial adhesive force of the gripper and the adhesive force after repeated manipulation, respectively.

**Table S1.** Material properties of the simulation model.

| Part | Material property | |
| --- | --- | --- |
|  | Constitutive model | Constant |
| Stainless steel sheet | Linear elastic model | *E* = 190 GPa, *v* = 0.305 |
| Adhesive film | Mooney Rivlin model | C10 = 0.201, C01 = 0.041, D1 = 0.0019 |
| Glass substrate | Linear elastic model | *E* = 55 GPa, *v* = 0.25 |

**Table S2.** Parameters for the analytical model.The adhesive strength of 150 kPa for the adhesive film is based on our previous research[60]. The thermodynamic work of adhesion of 45 mJ·m-2 is taken from references [78, 79]. The approximate initial crack length of 2.5 mm is obtained from the CCD monitoring in Fig. 2D and the FEA simulation in Fig. 2F.

| Parameter | Value |
| --- | --- |
| Elastic modulus of the stainless steel sheet *E*/GPa | 190 |
| Thickness of the stainless steel sheet *h*/mm | 0.2–0.53 |
| End length of the stainless steel sheet *a*/mm | 3 |
| Width of the adhesion module *b*/mm | 8 |
| Adhesion strength *Prange*/kPa | 150 |
| Thermodynamic work of adhesion *Wad*/mJ·m-2 | 45 |
| Length of the adhesive film *l*/mm | 10 |
| Length of the initial crack *lcrack0*/mm | ~2.5 |

Legends for movies

Movie S1: Peeling process with initial pulling angle of 90° captured by CCD camera.

Movie S2: Simulation animation of the peeling process with the initial pulling angle of 90°.

Movie S3: Dynamic attachment/detachment behavior of the adhesion module with a *θB* of 5.24° captured by CCD camera.

Movie S4: Simulation animation of the bending process with the initial pulling angle of 39.53°.

Movie S5: Application of the gripper in rapid handling of a thin glass substrate.

Movie S6: Application of the gripper in rapid handling of a thick glass substrate.

Movie S7: Application of the gripper in rapid handling of a 4-inch silicon wafer.

Movie S8: Application of the gripper in rapid handling of a PET film.

Movie S9: Release process on the millisecond scale (267 ms).
